# LINC complex protein nesprin-2 has pro-apoptotic activity via Bcl-2 family proteins

**DOI:** 10.1038/s41420-023-01763-w

**Published:** 2024-01-15

**Authors:** Liora Lindenboim, Hila Zohar, Gregg G. Gundersen, Howard J. Worman, Reuven Stein

**Affiliations:** 1 Department of Neurobiology, School of Neurobiology, Biochemistry and Biophysics, George S. Wise Faculty of Life Sciences, Tel Aviv University, Tel Aviv, 69978 Israel; 2 Department of Pathology and Cell Biology, Vagelos College of Physicians and Surgeons, Columbia University, New York, NY 10032 USA; 3 Department of Medicine, Vagelos College of Physicians and Surgeons, Columbia University, New York, NY 10032 USA

**Keywords:** Cell biology, Cell death

## Abstract

The apoptotic intrinsic pathway is initiated by perforation of the mitochondrial outer membrane by the effector pro-apoptotic proteins of the Bcl-2 family, Bax and Bak. Bax and Bak need to be activated, a process facilitated by the action of BH3-only pro-apoptotic members of the Bcl-2 family. The latter either directly activates the effector proteins or antagonizes the action of pro-survival Bcl-2 family members such as Bcl-x_L_. The nuclear envelope is a known target of the apoptotic machinery; however, it may also act as mediator of apoptosis. We showed previously that the nuclear envelope protein nesprin-2, a component of the linker of nucleoskeleton and cytoskeleton (LINC) complex, can bind to Bax in close proximity to the mitochondria and that the binding increases in apoptotic cells. We now show that depleting nesprin-2 inhibits the apoptotic mitochondrial pathway as measured by Bax and Bak activation and cytochrome c release. This survival effect was Bcl-x_L_-dependent. Nesprin-2 depletion also inhibited spontaneous exposure of the N-terminus of Bak in cells lacking Bcl-x_L_ and increased the presence of Bcl-x_L_ and Bax in the mitochondria. These results indicate that nesprin-2 promotes Bak activation and regulates mitochondrial translocation/retrotranslocation of Bcl-2 family proteins. Our findings demonstrate a new apoptotic pathway whereby the nuclear envelope, via nesprin-2, regulates apoptosis.

## Introduction

The nucleus, the largest and stiffest cellular organelle, is separated from the cytoplasm by the nuclear envelope (NE). However, the nucleus is physically connected to the cytoplasm through the cytoskeleton. This connection is mediated via the linker of nucleoskeleton and cytoskeleton (LINC) complex which is composed of Klarsicht/ANC-1/Syne-1 homology (KASH) domain proteins (known as nesprins in mammals) in the outer nuclear membrane and SUN (Sad1/UNC-84) domain proteins in the inner nuclear membrane [1–5]. The KASH domain of nesprins projects into the perinuclear space, where it interacts with the SUN domain of proteins such as SUN1 and SUN2. The N-termini of nesprins extend into the cytoplasm where they interact with three major cytoskeletal elements: actin microfilaments, intermediate filaments, and microtubules, thus connecting the cytoskeleton to the SUN proteins in the inner nuclear membrane. SUN proteins in turn interact with A-type lamins, chromatin-binding proteins, and other nuclear proteins [1].

In mammals, there are six KASH domain proteins. Two of them, nesprin-1 and nesprin-2, are encoded by genes containing more than 100 exons that lead to multiple isoforms [6, 7]. The largest isoforms of nesprin-1 and nesprin-2 are termed nesprin-1-giant and nesprin-2-giant (nesprin-2G), respectively. These proteins have an N-terminal actin-binding site consisting of paired actin-binding calponin-homology (CH) domains, followed by a rod-like structure composed of multiple spectrin-repeats. Binding of nesprin-2G to actin is also facilitated by interactions with FHOD1 [8, 9] and fascin [10]. There are also nesprin-2 isoforms that lack the actin-binding domains. Some of them as well as nesprin-2G interact with microtubules via a carboxyl terminal region that bind to kinesin-1 and cytoplasmic dynein [11].

Apoptosis is a regulated process which is mediated by sequential events that culminate in cell death. Two main protein families regulate and execute the cell death process: Bcl-2 proteins and caspases. Bcl-2 proteins act mainly, but not only, by regulating the integrity of mitochondria. Caspases are cysteine aspartate proteases that cleave a subset of essential cellular proteins to promote apoptotic cell death. The cell nucleus and its NE are a well-defined target in apoptosis, with their proteins degraded by caspases. However, the nucleus and the NE may not only be targets but also act as mediators of apoptosis (for review see [12] and references therein).

There are two functional groups of Bcl-2 proteins: pro-survival and pro-apoptotic (for review see [13–18]). The pro-apoptotic group is comprised of the effector proteins Bax and Bak as well as the Bcl-2 homology domain 3 (BH3)-only proteins such as Bid, Bim, and Bad. The pro-survival proteins, such as Bcl-2 and Bcl-x_L_, bind to and block the activity of the pro-apoptotic proteins. Bax and Bak promote mitochondrial outer membrane permeabilization (MOMP) and release of apoptogenic factors such as cytochrome *c* from the mitochondrial intermembrane space. This leads to caspase activation via the apoptosome [19]. Pro-survival Bcl-2 proteins inhibit MOMP by binding directly to BH3-only proteins or by binding to activated Bax and Bak.

Bcl-2 family proteins also have non-apoptotic [20–22] or non-canonical functions. Accordingly, we showed previously that in response to apoptotic stimuli Bax triggers nuclear protein redistribution (NPR) [23, 24]. This process involves Bax-regulated disturbances in NE proteins, including lamin A/C, which may result in the generation and subsequent rupture of nuclear protein-containing bubbles encapsulated by nuclear pore complex-depleted nuclear membranes. We termed the later “stress-induced generation and rupture of nuclear bubbles” (SIGRUNB) [25]. These Bax-regulated effects may involve Bax-dependent impairment of the integrity of the LINC complex as apoptotic stimuli induce Bax/Bak-dependent, but caspase-independent, subcellular redistribution of nesprin-1 and nesprin-2. Moreover, nesprin-2 interacts with Bax in close proximity to perinuclear mitochondria and Bax/nesprin-2 interaction increases during apoptosis [26]. Given these results we hypothesized that nesprin-2 may play a role in regulating the apoptotic process. Here, we test this hypothesis and show that depleting nesprin-2 from mouse embryonic fibroblasts (MEFs) inhibits apoptosis-induced Bax and Bak activation and the resulting MOMP, indicating that nesprin-2 has pro-apoptotic activity. Furthermore, the results imply that the nesprin-2 apoptotic effect is mediated by inhibiting the anti-apoptotic activities of Bcl-x_L_.

## Results

### Depletion of nesprin-2 inhibits apoptotic cell death

**Fig. 1:**
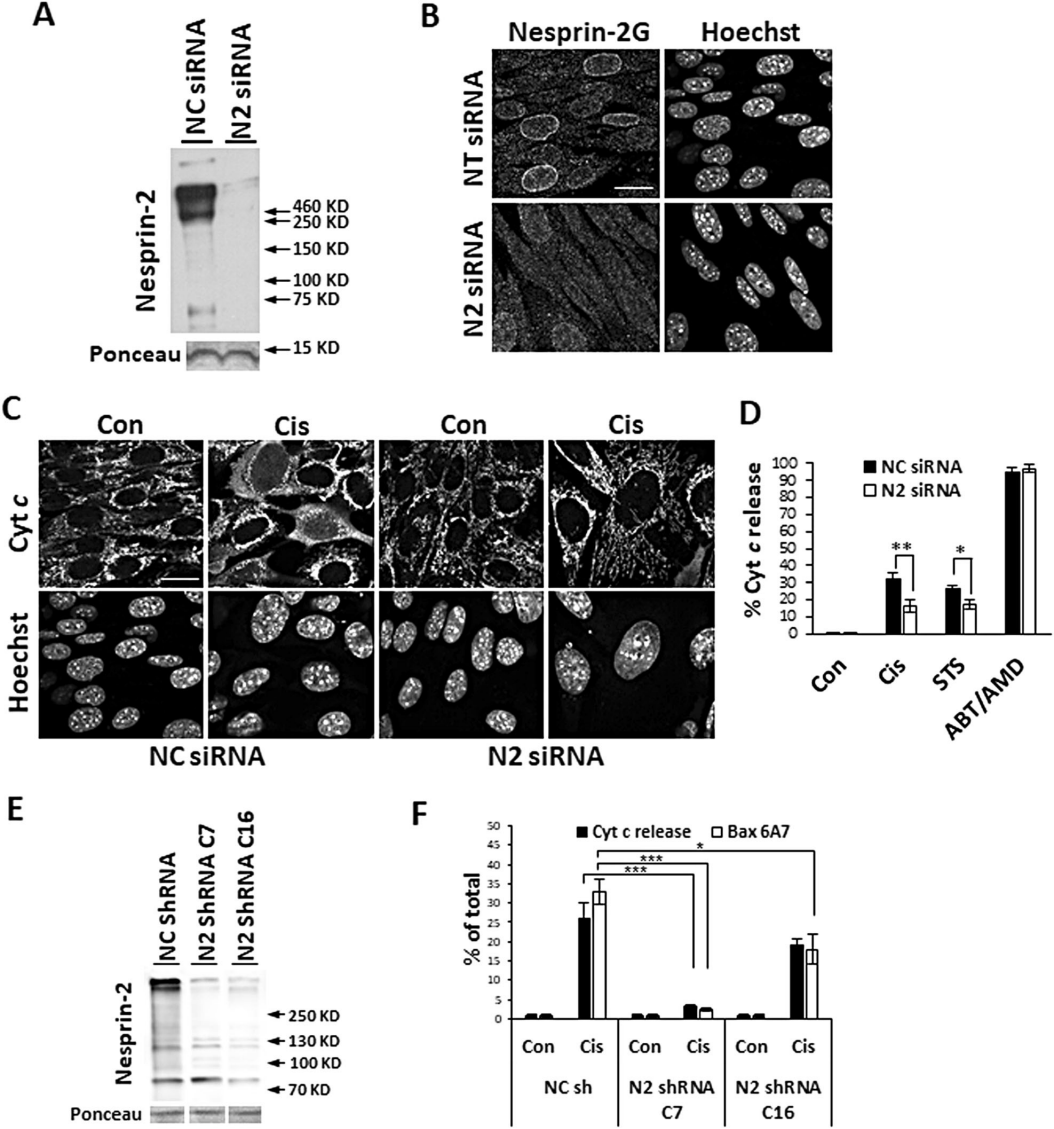
Knockdown of nesprin-2 inhibits cell death. **A**–**D** Knockdown of nesprin-2 using siRNA. **A** A representative immunoblot (from *n* = 5) of lysates of WT MEFs transfected with non-targeting [negative control (NC)] siRNA (NC siRNA) or nesprin-2 siRNA (N2 siRNA) for 72 h probed with anti-nesprin-2 K2 Ab. Ponceau staining was used as internal loading control. **B** Representative confocal IF micrographs of WT MEFs transfected with NC siRNA or N2 siRNA (*n* = 3 and 10 respectively) stained with anti-nesprin-2G Ab and Hoechst dye (to detect nuclei). The IF micrographs show the same field visualized separately for nesprin-2G Ab labeling and nuclei staining. Bar = 20 µm. **C**, **D** Apoptotic cell death assessed by cytochrome *c* (Cyt *c*) release. WT MEFs were treated three days after siRNA transfection for 24 h with cisplatin (Cis), staurosporine (STS) or ABT-737 plus actinomycin D (ABT/AMD) together with Q-VD-OPH or with no drug (Con). **C** Representative confocal IF micrographs of cisplatin-treated WT MEFs labeled with anti-cytochrome *c* Ab (Cyt *c*) and Hoechst dye. Bar = 20 µm. **D** Quantification of the percentage of cells exhibiting cytochrome *c* release. The results are expressed as percentage of cells exhibiting cytochrome *c* release from the total population (at least 100 cells). Values are presented as mean ± SEM (error bars). *p < 0.05, **p < 0.01; two-way ANOVA followed by two tailed student’s *t*-test; *n* = 5. **E**, **F** Nespri*n*-2 knockdown by nesprin-2 shRNA (N2 shRNA). **E** A representative immunoblot of nesprin-2 in the negative control (NC shRNA) and nesprin-2 shRNA (N2 shRNA) clones C16 (N2 shRNA C16) and clones C7 (N2 shRNA C7) (*n* = 4 and 3 for nespri*n*-2 shRNA clones C7 and C16, respectively). Nesprin-2 was detected using anti-nesprin-2 K2 Ab. Ponceau staining was used as internal loading control. **F** Nesprin-2 knockdown reduced cisplatin-induced cytochrome *c* release and Bax activation [detected using the anti-Bax Ab 6A7 (Bax 6A7)]. Negative control or nesprin-2 clones were untreated (Con) or treated for 24 h with cisplatin (Cis) and Q-VD-OPH. The results shown are quantification of the IF staining and are expressed as the percentage of cells exhibiting cytochrome *c* release or Bax NT exposure from total population (at least 100 cells). Values are presented as mean ± SEM (error bars) (*n* = 3). (*p < 0.05, ***p = 0.001; One-way ANOVA followed by Dunnett’s multiple comparisons test for each cytochrome *c* release or Bax 6A7).

To examine the effect of nesprin-2 on apoptosis we assessed the outcome of its depletion on cell death. We transfected WT MEFs with a pool of four nesprin-2 siRNAs (herein nesprin-2 siRNA) that target exons 92, 93, 95, 102, and 103 of *Syne2* and are expected to target most if not all nesprin-2 isoforms. This treatment substantially decreased nesprin-2 expression 72 h post-transfection as indicated by immunoblot analysis, which showed depletion of high and low MW nesprin-2 bands (Fig. 1A), and by IF microscopy, which showed disappearance of NE-associated nesprin-2G (Fig. 1B). After depleting nesprin-2, the cells were treated with the apoptotic stimuli cisplatin, staurosporine or ABT-737 plus actinomycin D (AMD) [the latter promotes apoptosis by inhibiting the pro-survival proteins Bcl-x_L_, Bcl-2 and Bcl-w (by ABT-737) and Mcl-1 by AMD], and the effect of nesprin-2 siRNA on apoptosis was compared to that of control non-targeting siRNA. We used MOMP (indicated by cytochrome *c* release) as an apoptotic readout. To increase the number of cells available for the analysis, cell death was attenuated by treating the cells with the pan-caspase inhibitor Q-VD-OPH. The results (Fig. 1C and D and Supplementary Fig. S1A) show that nesprin-2 siRNA significantly reduced cytochrome *c* release induced by cisplatin and staurosporine, but not by ABT-737/AMD, compared to non-targeting siRNA. To exclude the possibility that the nesprin-2 siRNA effect on cell death was mediated by off-target effects, we depleted nesprin-2 using a shRNA corresponding to a sequence at the 3′-UTR [27] of *Syne2* that targets a different location than the siRNAs. This nesprin-2 shRNA has been shown to deplete nesprin-2G and other nesprin-2 isoforms [27]. WT MEFs were infected with a retrovirus directing expression of the nesprin-2 shRNA [27]. Two independent clones (C7 and C16) had stable knockdown of nesprin-2 as assessed by immunoblot (Fig. 1E) and IF microscopy (Fig. S1B). Apoptotic death was monitored by Bax activation indicted by its NT exposure, recognized by the anti-Bax 6A7 Ab [28], and by cytochrome *c* release (Fig. S1C). Both Bax activation and cytochrome *c* release were significantly lower in cisplatin-treated C7 MEFs compared to control (Fig. 1F). Similar results were obtained using C16 MEFs, although the extent of the reduction of apoptosis was lower than in the C7 clone and did not reach significance for cytochrome *c* release (Fig. 1F). These results show that depleting nesprin-2 by two different approaches increases the resistance of WT MEFs to apoptosis, thus implying that nesprin-2 has a pro-apoptotic activity.

**Fig. 2:**
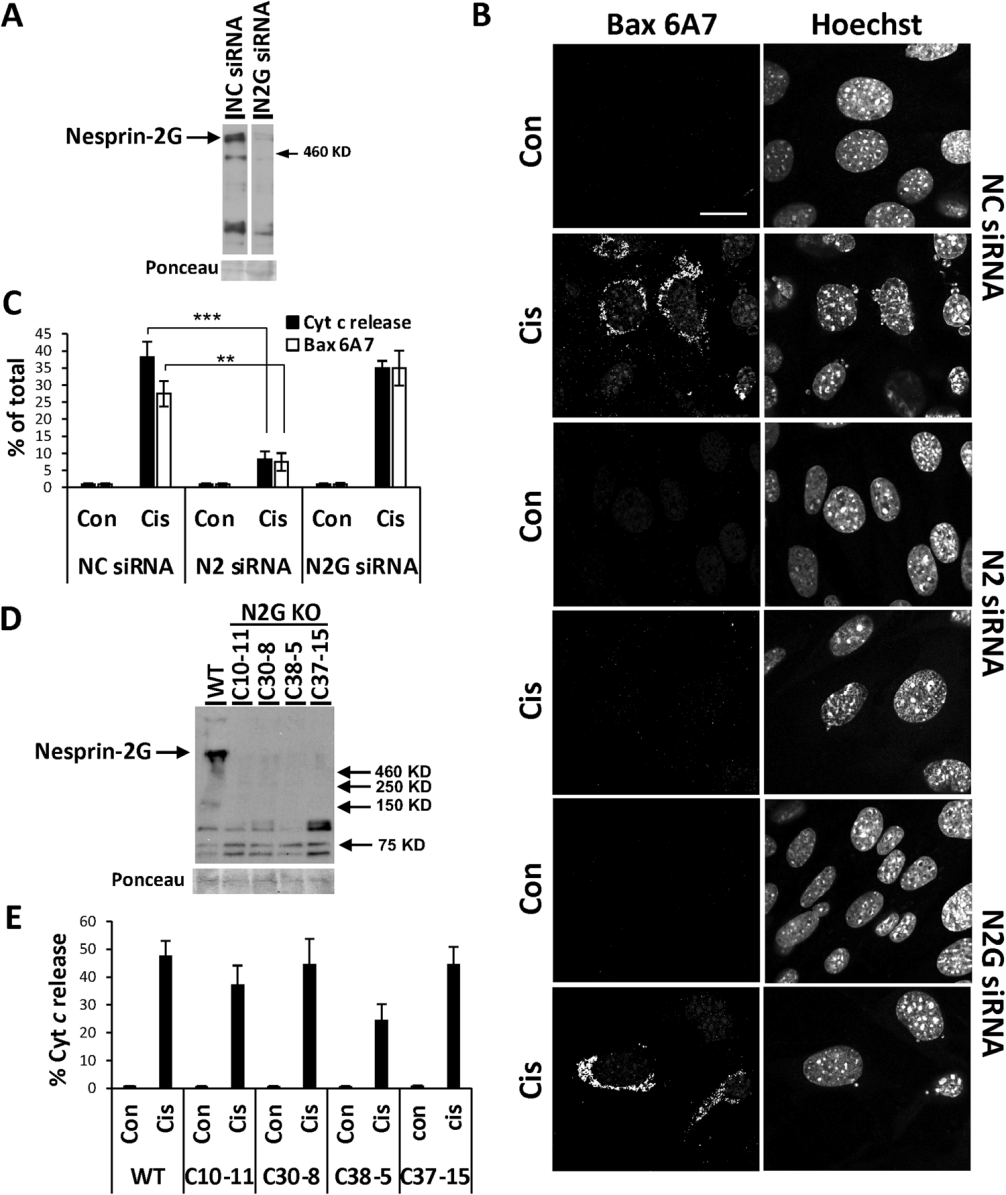
Nesprin-2G depletion does not affect apoptotic cell death. **A** The effect of nesprin-2G knockdown on nesprin-2 expression in WT MEFs transfected with non-targeting [negative control (NC)] siRNA (NC siRNA) or nesprin-2G (N2G) siRNAs for 72 h. The results show a representative immunoblot (from *n* = 2) of lysate probed with nesprin-2 K2 Ab. Ponceau staining was used as an internal loading control. **B**, **C** Death of WT MEFs treated three days after transfection with the indicated siRNAs for 24 h with no drug (Con) or cisplatin (Cis) and Q-VD-OPH. **B** Representative (*n* = 4) confocal IF micrographs of WT MEFs labeled with anti-Bax 6A7 Ab and Hoechst dye. Bar = 20 µm. **C** Quantification of cytochrome *c* (Cyt *c*) release and Bax NT exposure (Bax 6A7) determined from IF micrograph of WT MEFs labeled with Bax 6A7 (**B**) or anti-cytochrome *c* (Cyt c) (shown in Supplementary Fig. S2B) in the indicated treatments. The results are expressed as percentage of cells exhibiting cytochrome *c* release or Bax 6A7 staining from total population (at least 100 cells). Values are presented as mean ± SEM (error bars) (*n* = 4). (**p < 0.01, ***p < 0.001; One-way ANOVA followed by Dunnett’s multiple comparisons test for each cytochrome *c* release or Bax 6A7). **D**, **E** The effect of nesprin-2G knockout. **D** A representative immunoblot of nesprin-2G expression in the indicated four nesprin-2G knockout (N2G KO) clones. WT MEFs (WT) were used as positive control and Ponceau staining as an internal loading control. **E** The effect of nesprin-2G knockout on cisplatin-induced cytochrome *c* release. WT MEFs and the different nesprin-2 knockout clones were treated and analyzed for cytochrome *c* (Cyt *c*) release as described in **B**, **C** (*n* = 3 and 5 for C30-8 and other clones, respectively).

The nesprin-2 siRNA and shRNA are expected to target nesprin-2G as well as other isoforms. To examine if nesprin-2G is sufficient to promote the apoptotic effect, we depleted it from WT MEFs using siRNA targeting the amino terminus of nesprin-2G. This siRNA knocked down nesprin-2G expression (Fig. 2A and Supplementary Fig. S2A, immunoblotting and IF microscopy respectively) but did not affect cisplatin-induced apoptotic cell death monitored by Bax activation (Fig. 2B and C) and cytochrome *c* release (Fig. 2C and Supplementary Fig. S2B). To further assess the effect of nesprin-2G we also generated four independent clones by CRISPR/Cas9 in which a portion of *Syne2* encoding nesprin-2G was deleted and demonstrated absence of the protein by immunoblotting (Fig. 2D) and IF microscopy (Fig. S2C). In three of these clones, cytochrome *c* release was similar to that of WT MEFs whereas in clone C35-8 it was lower (Figs. 2E and S2D). However, one-way ANOVA of WT and the four clones did not show significant difference between them. These results suggest that depletion of nesprin-2G alone is not sufficient to promote cell survival.

### Nesprin-2 depletion does not affect other LINC complex or apoptotic proteins levels

**Fig. 3:**
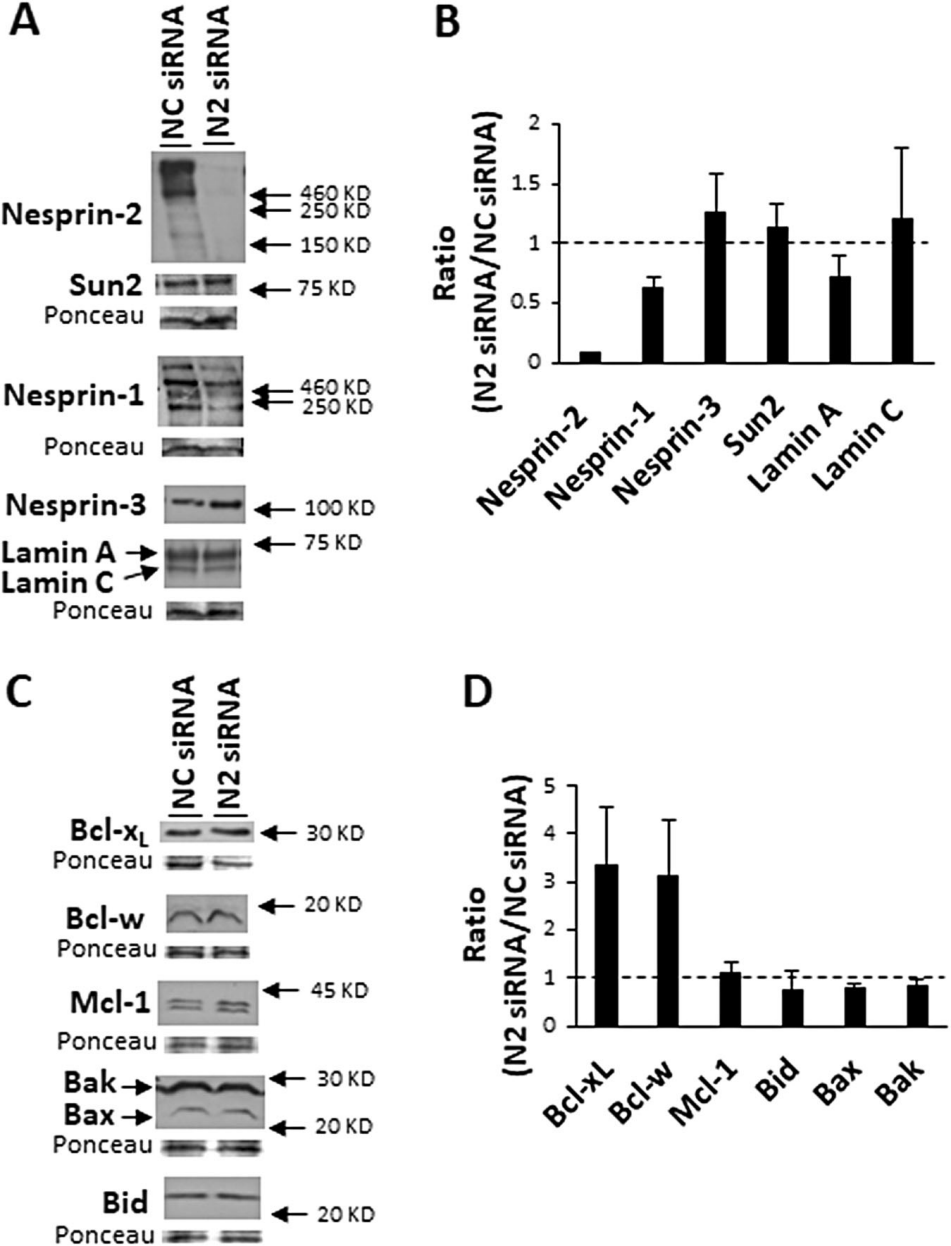
Nesprin-2 depletion does not affect the expression of LINC complex and Bcl-2 family proteins. Expression level of LINC complex and Lamin A/C proteins (**A**, **B**) or Bcl-2 family proteins (**C**, **D**) in WT MEFs transfected with non-targeting [negative control (NC)] or nesprin-2 (N2) siRNA for 72 h. **A** and **C** show representative immunoblots of lysates probed for the indicated proteins. Ponceau staining was used as internal loading control. **B** and **D** show quantification of the expression levels of the different proteins from all experiments. Protein levels were normalized to internal loading control as described in the “Materials and Methods” section and the results are expressed as the ratio of the normalized protein level in the nesprin-2 (N2) siRNA treatment relative to non-targeting [negative control (NC)] siRNA (NC siRNA) treatment and are presented as mean ± SEM (error bars) (*n* = 3 for LINC complex proteins, *n* = 5 for Mcl-1 and Bid; Bax, Bak and Bcl-w and *n* = 9 for Bcl-x_L_). No significance difference in protein expression between negative control and nesprin-2 depleted cells was observed for all proteins, besides for nesprin-2 (*p* < 0.0001). (One-sample *t*-test. Dashed line: ratio = 1).

To unravel the mechanism whereby nesprin-2 depletion promotes survival, we first examined if such an effect involves changes in the level of other LINC complex proteins or apoptotic proteins. We examined the effect of nesprin-2 siRNA on the LINC complex proteins nesprin-1, nesprin-3, SUN2, and the LINC complex associated proteins lamin A/C. For apoptotic proteins, we focused on the Bcl-2 family, since the nesprin-2 depletion survival effect inhibits Bax activation and MOMP and nesprin-2 binds to Bax [26]. WT MEFs were treated with nesprin-2 or non-targeting siRNA and proteins expression was assessed by immunoblotting. While nesprin-2 level was substantially reduced, the levels of the other LINC complex proteins did not change significantly (Fig. 3A and B) consistent with earlier results [29]. There was about 40% reduction in nesprin-1 protein level, however it did not reach significance. Nesprin-2 knockdown also did not significantly affect the expression level of the Bcl-2 family proteins, neither of the pro-apoptotic proteins Bax, Bak, and Bid, nor the anti-apoptotic protein Mcl-1. The level of Bcl-x_L,_ and Bcl-w was increased, however it did not reach significance (Fig. 3C and D). These results indicate that the survival effect of lack of nesprin-2 is not mediated by affecting the expression level of neither other LINC complex proteins, nor Bcl-2 family proteins.

### Nesprin-2 displacement from the NE per se does not affect apoptosis

**Fig. 4:**
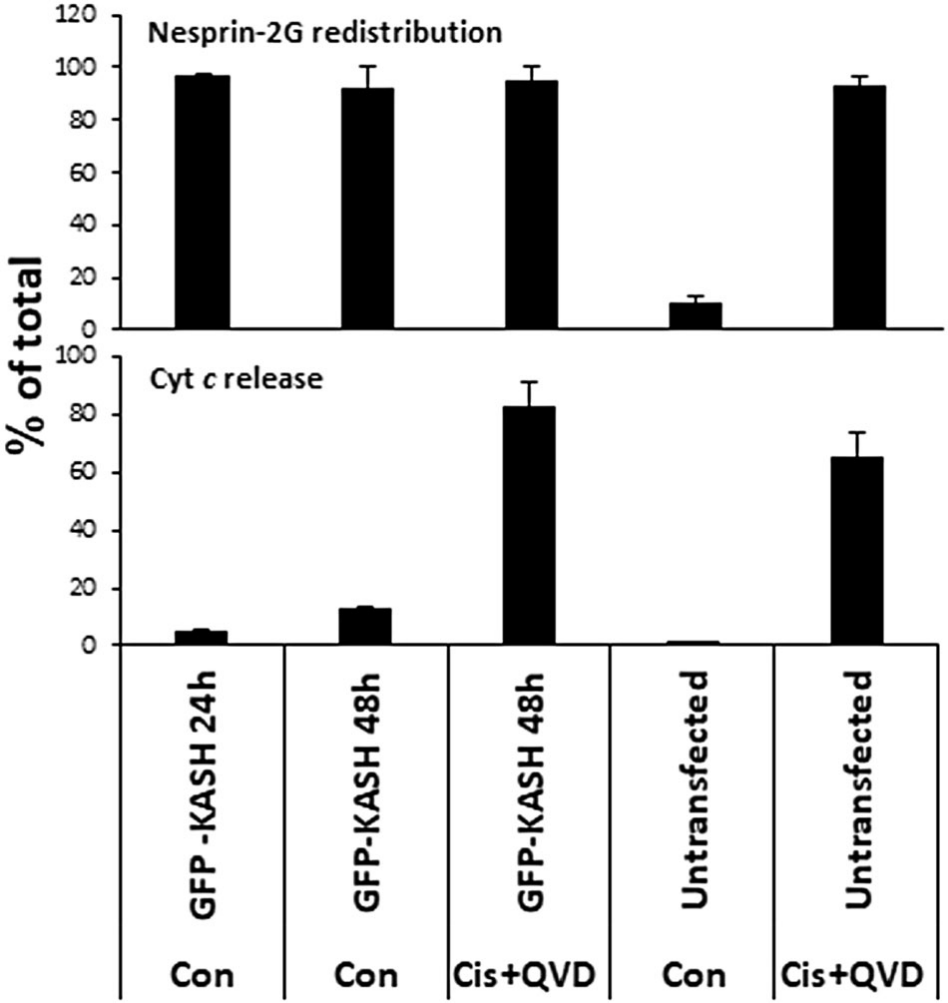
Displacement of nesprin-2 from the NE by GFP-KASH does not induce cell death nor inhibit cis-induced cell death. WT MEFs were untransfected or transfected with a GFP-KASH expression vector for 24 h followed by treatment with no drug (Con) or with cisplatin (Cis) and Q-VD-OPH for an additional 24 h (total 48 after transfection), after which they were IF stained for cytochrome *c* (Cyt *c*) and nesprin-2G (the corresponding images are shown in Supplementary Fig. S3). The results shown are quantification of the percentage of cells exhibiting nesprin-2G redistribution (upper panel) and cytochrome *c* release (lower panel) in each treatment from the total population (at least 100 cells). Values are presented as mean ± SEM (error bars) (*n* = 3).

Nesprin-2 depletion reduces a major element in mechanotransduction to the nucleus [29, 30]. To test whether the inhibition of apoptosis by nesprin-2 depletion is due to the reduction in mechnotransduction or the loss of nesprin-2 as a molecular species, we tested whether apoptosis is blocked by expression of dominant negative GFP-KASH, which displaces endogenous nesprin-2 from the NE (by disrupting its interaction with SUN proteins) and reduces mechnotransduction [31]. WT MEFs were transfected with GFP-KASH, and 24 h later, when nesprin-2 was dislodged from the vast majority (96%) of the transfected cells NE (Fig. 4 upper panel and Supplementary Fig. S3), the cells were treated with cisplatin for additional 24 h and then cell death was monitored by assessing cytochrome *c* release. Redistribution of nesprin-2 from the NE before exposing the cells to the apoptotic trigger did not inhibit cisplatin-induced cytochrome *c* release (Fig. 4 lower panel and Supplementary Fig. S3). This result indicates that the survival effect caused by lack of nesprin-2 is not mediated by the absence of nesprin-2 from the NE.

The results also show that GFP-KASH expression (without cisplatin) for 24 or 48 h did not induce cell death (Fig. 4 and Supplementary Fig. S3), suggesting that nesprin-2 redistribution from the NE in itself is insufficient to induce apoptotic cell death.

### The effect of nesprin-2 depletion on cell survival does not involve inhibition of NPR

**Fig. 5:**
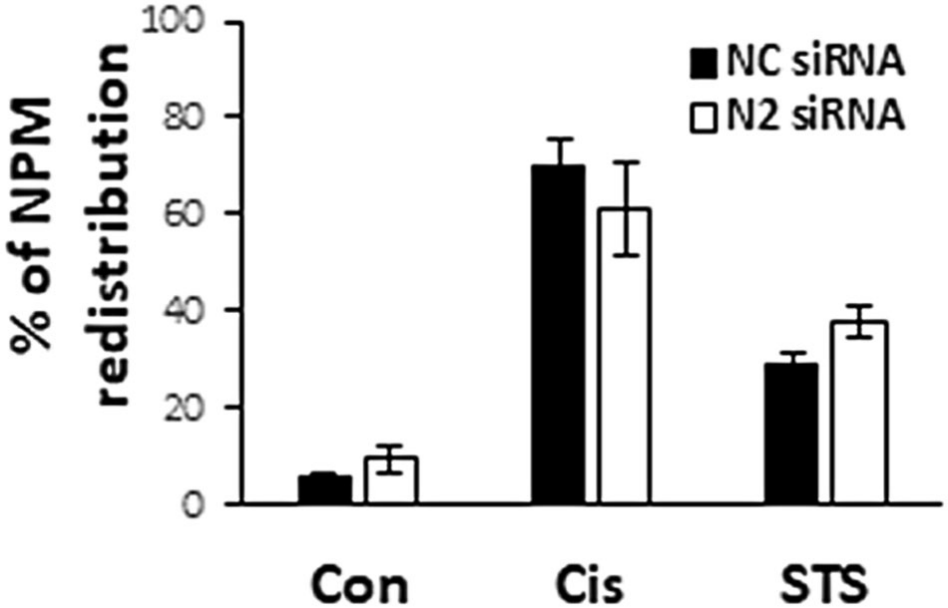
Nesprin-2 depletion does not affect NPR. WT MEFs were transfected with non-targeting [negative control (NC)] or nesprin-2 (N2) siRNA and treated with no drug (Con) treated with cisplatin (Cis) or staurosporine (STS) and QVD-OPH as described in Fig. 1A. Then, the cells were IF stained with anti-nucleophosmin (NPM) Ab and visualized by fluorescence microscopy (the corresponding images are shown in Supplementary Fig. S4). The results show quantification of nucleophosmin (NPM) redistribution (appearance of nucleophosmin in the cytoplasm) in each treatment and are expressed as the percentage of cells exhibiting nucleophosmin redistribution from the total cell population (at least 100 cells). Values are presented as mean ± SEM (error bars) (*n* = d3).

Previously, we showed that apoptotic triggers lead to Bax/Bak-dependent NPR [24]. Other studies showed that redistribution of some nuclear proteins may prompt cell death [32]. We therefore examined whether the survival effect mediated by nesprin-2 depletion involves inhibition of NPR. We treated WT MEFs with non-targeting or nesprin-2 siRNA, followed by cisplatin or staurosporine treatment. As expected, both apoptotic stimuli induced NPR, as indicated by redistribution of the nuclear protein nucleophosmin [23]. However, depletion of nesprin-2 did not affect nucleophosmin redistribution induced by either cisplatin or staurosporine (Fig. 5 and Supplementary Fig. S4). These results suggest that inhibition of NPR does not play a role in the effect of nesprin-2 depletion on cell survival. Furthermore, the results indicate that nesprin-2 is not required for NPR.

### Nesprin-2 depletion promotes survival by Bcl-x_L_-dependent manner and inhibits Bak-NT exposure independently of Bcl-x_L_

**Fig. 6:**
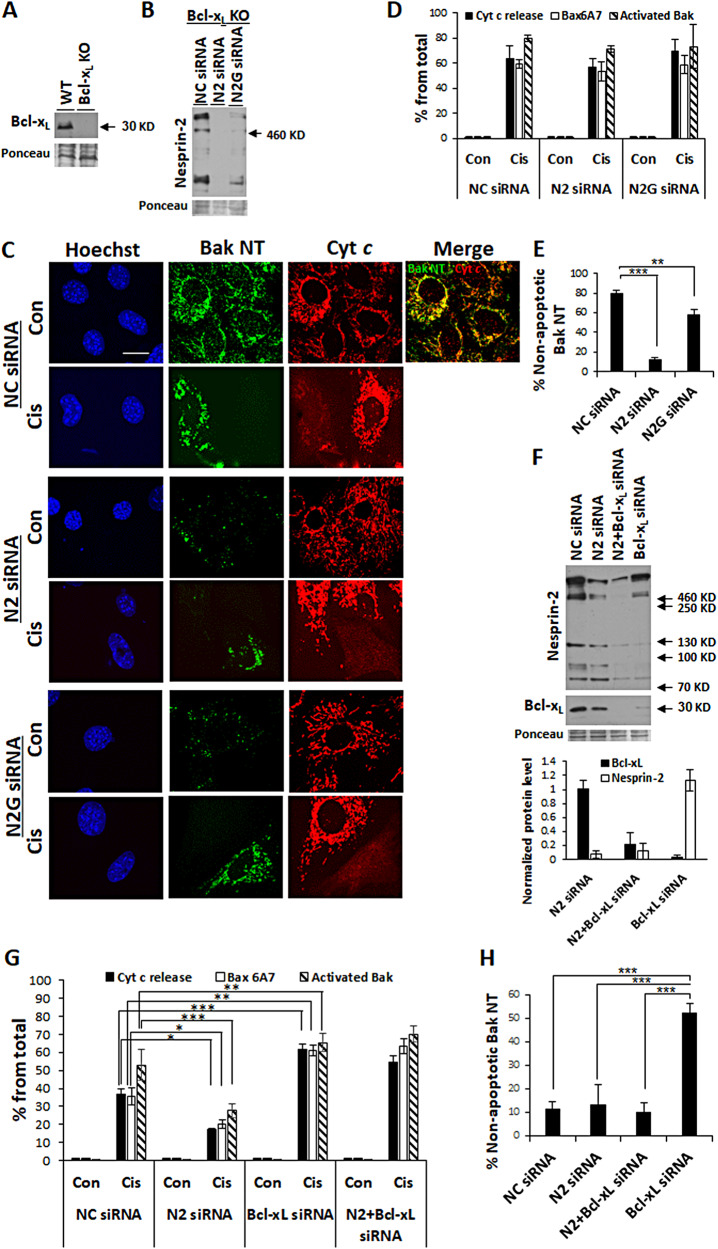
The effect of lack of Bcl-x_L_ on the nesprin-2 depletion survival effect. **A**–**E** The effects of nesprin-2 depletion in Bcl-x_L_ KO MEFs. **A**, **B** Representative immunoblots (from *n* = 2) of lysates of WT and Bcl-x_L_ KO MEFs probed with anti-Bcl-x_L_ Ab (**A**) or of Bcl-x_L_ KO MEFs transfected with non-targeting [negative control (NC)], nesprin-2 (N2) or nesprin-2G (N2G) siRNAs for 72 h probed with anti-nesprin-2 K2 Ab (**B**). Ponceau staining was used as internal loading control. **C**, **D** Apoptotic events in Bcl-x_L_ KO MEFs treated three days after siRNAs transfection for 24 h with no drug (Con) or cisplatin (Cis) and Q-VD-OPH and labeled by anti-cytochrome *c*, anti-Bax 6A7 (shown in Supplementary Fig. S5C) or anti-Bak NT Ab and Hoechst dye and visualized by confocal fluorescence microscopy. Bar = 20 µm. **C** Representative (*n* = 4) confocal IF micrographs of Bak NT, cytochrome *c* (Cyt c), and Hoechst staining. Bar = 20 µm A merged image of cells treated with NC siRNA without cisplatin is shown in the upper right end of the panel. **D** Quantification of the percentage of cells in each treatment exhibiting Bax activation (Bax 6A7), Bak activation (defined by appearance of strong punctuated perinuclear Bak-NT signal), or cytochrome *c* release, from total population (at least 100 cells). Values are presented as mean ± SEM (error bars) (*n* = 4). **E** Quantification of the percentage of cells exhibiting non-apoptotic Bak-NT signal (elongated staining pattern) in cells treated with the indicated siRNAs. The results are expressed as percentage of cells exhibiting Bak-NT signals from total population (at least 100 cells). Values are presented as mean ± SEM (error bars) (*n* = 5). (**p < 0.01, ***p < 0.001; One-way ANOVA followed by Dunnett’s multiple comparisons test). **F**–**H** The effects of nesprin-2 depletion in WT MEFs depleted of Bcl-x_L_. **F** Nesprin-2 expression in WT MEFs transfected with non-targeting [negative control (NC)] siRNA (NC siRNA), nesprin-2 (N2) siRNA, nesprin-2 and Bcl-x_L_ siRNAs (N2 + and Bcl-x_L_ siRNA) or Bcl-x_L_ siRNA for 72 h. The upper panel show a representative immunoblot of the indicated treatments probed with anti-nesprin-2 K2 or anti- Bcl-x_L_ Abs. Ponceau staining was used as internal loading control. The lower panel shows quantification of the nesprin-2 (nesprin-2 bands of molecular masses greater than 460 kDa) and Bcl-x_L_ expression levels, normalized to Ponceau bands. The results are presented as the normalized protein values in the indicated siRNA treatments, relative to non-targeting [negative control (NC)] siRNA (NC siRNA) and are expressed as mean ± SEM (error bars) (*n* = 3). **G** Effects on apoptosis. 72 h following siRNAs transfections cells treated with no drug (Con) or with cisplatin (Cis) and Q-VD-OPH were stained for the indicated apoptotic events and the percentage of cells exhibiting them was quantified and expressed as described in Fig. 6D. Values are presented as mean ± SEM (error bars). [*p < 0.05, **p < 0.01, ***p ≤ 0.001; One-way ANOVA followed by Dunnett’s multiple comparisons test for each cytochrome *c* release or Bax 6A7 (*n* = 3) and two-way ANOVA followed by Dunnett’s multiple comparisons test for activated Bak (*n* = 6)]. **H** Quantification of the effect of nesprin-2 depletion on non-apoptotic Bak-NT exposure. WT MEFs were transfected with the different siRNAs as described above and the percentage of cells exhibiting Bak-NT signal in the control cells (no drug) of the different siRNAs treatments was assessed. The results are expressed as percentage of cells exhibiting Bak-NT signal from total population (at least 100 cells). Values are presented as mean ± SEM (error bars) (*n* = 3). (***p < 0.001); One-way ANOVA followed by Dunnett’s multiple comparisons test between Bcl-x_L_ siRNA to non-targeting [negative control (NC)] siRNA, nesprin-2 siRNA or Bcl-x_L_ or nesprin-2 siRNAs.

Given that depletion of nesprin-2 inhibits Bax activation and MOMP, we hypothesized that this effect may involve the action of anti-apoptotic Bcl-2 family proteins such as Bcl-x_L_. To test this hypothesis, we examined the ability of nesprin-2 siRNA to inhibit cisplatin-induced MOMP in *Bcl-x*_*L*_^*−/−*^ (herein Bcl-x_L_ KO) MEFs. The effect of nesprin-2G siRNA was also examined. We first confirmed that Bcl-x_L_ KO MEFs do not express Bcl-x_L_ (Figs. 6A and S5A) and that nesprin-2 and nesprin-2G siRNAs reduced the expression of the corresponding proteins in these cells (Figs. 6B and S5B).

Given that Bak is also a known MOMP activator [33], in addition to Bax activation and cytochrome *c* release we also monitored cisplatin-induced Bak activation, assessed by IF staining using an antibody that recognizes the Bak N-terminus (NT). Bak activation is defined as Bak-NT exposure and distribution of Bak into enlarged puncta (see Fig. 6C). As expected, treatment with cisplatininduced Bax activation, Bak activation, and cytochrome *c* release in Bcl-x_L_ KO MEFs treated with non-targeting or nesprin-2G siRNAs. However, in contrast to its inhibitory effect in WT MEFs, nesprin-2 siRNA did not inhibit these cisplatin-induced apoptotic events in Bcl-x_L_ KO MEFs (Fig. 6C and D and Supplementary Fig. S5C). Hence, Bcl-x_L_ is needed for the anti-apoptotic effect of nesprin-2 depletion. These results further indicate that nesprin-2 promotes apoptosis by acting through Bcl-x_L_, probably by inhibiting Bcl-x_L_ anti-apoptotic activity.

In healthy cells BAK is dormant and mainly resides in the mitochondria [34, 35]. We noticed that in untreated healthy Bcl-x_L_ KO MEFs, Bak-NT antibody detected Bak-NT signal that was localized in mitochondria, as indicated by its spaghetti-like staining pattern and co-localization with cytochrome *c* (Fig. 6C, upper panel). This Bak-NT signal probably represents Bak molecules that have undergone partial activation (Bak-NT exposure) owing to the lack of Bcl-x_L_, but did not mature to fully activated Bak molecules. Notably, the Bak-NT exposure in healthy Bcl-x_L_ KO MEFs was inhibited by treatment with nesprin-2 or nesprin-2G siRNAs, although the latter to a lesser extent (Fig. 6E). This result suggests that nesprin-2 regulates Bak activation also independently of Bcl-x_L_.

To further confirm the results obtained using Bcl-x_L_ KO MEFs, we assessed the effect of nesprin-2 depletion in WT MEFs with Bcl-x_L_ expression reduced by siRNA. Bcl-x_L_ or nesprin-2 siRNA inhibited Bcl-x_L_ or nesprin-2 expression, either when applied alone or in combination with each other (Figs. 6F and S5D and E). Cisplatin treatment induced Bax and Bak activation and cytochrome *c* release in WT MEFs treated with negative control or Bcl-x_L_ siRNA. As expected from cells lacking Bcl-x_L,_ these apoptotic effects were significantly higher in the Bcl-x_L_ siRNA-treated cells (Figs. 6G and S5F and G). Nesprin-2 siRNA significantly inhibited cisplatin-induced Bax and Bak activation and cytochrome *c* release. However, nesprin-2 siRNA did not inhibit these cisplatin-induced apoptotic events when applied together with Bcl-x_L_ siRNA (Figs. 6G and S5F and G). Notably, knockdown of Bcl-x_L_ in cisplatin-untreated WT MEFs induced Bak-NT exposure and this effect was significantly inhibited by nesprin-2 siRNA (Fig. 6H and Supplementary Fig. S5G). Taken together the results obtained using Bcl-x_L_ KO MEFs and WT MEFs depleted of Bcl-x_L_ suggest that the pro-apoptotic activity of nesprin-2 is mediated by inhibiting the anti-apoptotic effects of Bcl-x_L_ and by promoting Bak-NT exposure independently of Bcl-x_L_.

### Nesprin-2 depletion increases the mitochondrial portion of Bcl-x_L_ and Bax

**Fig. 7:**
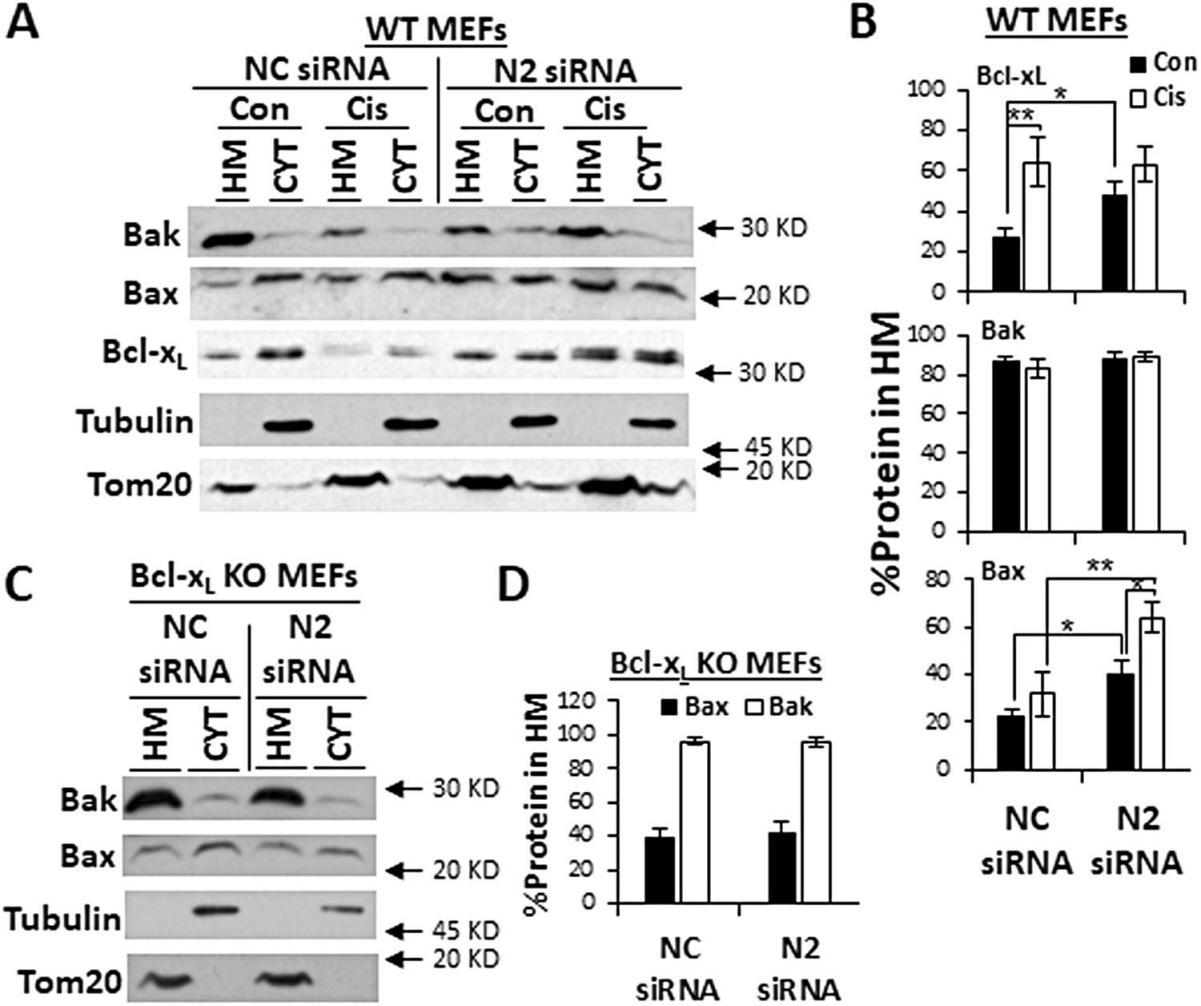
Nesprin-2 depletion increases the portion of mitochondrial Bcl-x_L_ and Bax in WT, but not in Bcl-x_L_ KO MEFs. **A**, **B** The effect of nesprin-2 depletion on subcellular localization of Bak, Bax, and Bcl-x_L_ in WT MEFs. WT MEFs treated three days after transfection with non-targeting negative control (NC) or nesprin-2 (N2) siRNA for 24 h with no drug (Con), or with cisplatin (Cis) and Q-VD-OPH were subjected to subcellular fractionation as described in Materials and Methods. The presence of Bak, Bax, and Bcl-x_L_ in cytosolic (CYT) and heavy membrane (HM) fractions was determined by immunoblotting using the corresponding Abs. The purity of the fractions was assessed by the presence of the cytosolic protein tubulin and the mitochondrial protein Tom20 in the different subcellular fractions. **A** Representative immunoblots probed for the indicated proteins. **B** Quantification of the expression levels of Bcl-x_L_, Bak, and Bax in the different siRNA treatments. The results are presented as the percentage of each protein in the HM fraction from total (HM + CYT) and are expressed as mean ± SEM (error bars) (*n* = 8 and 4 for untreated and cisplatin-treated cells, respectively). *q < 0.05, **q < 0.01, ***q < 0.001; Two-way ANOVA followed by simple main effect tests corrected for multiple comparisons using the False Discovery Rate method. **C**, **D** The effect of nesprin-2 depletion on subcellular localization of Bak and Bax in Bcl-x_L_ KO MEFs. Bcl-x_L_ KO MEFs were transfected with non-targeting negative control (NC) or nesprin-2 (N2) siRNA. After 72 h subcellular fractionation was performed and the presence of Bak and Bax in each fraction was determined by immunoblotting as described above. **C** The corresponding representative immunoblots and the quantification (**D**) of the expression levels of Bax and Bak in the indicated treatments was performed presented and expressed as described above (*n* = 4).

The results showing that nesprin-2 may act as an inhibitor of Bcl-x_L_ prompted us to examine if its depletion can affect translocation/retrotranslocation of Bcl-2 family proteins to the mitochondria. We transfected WT MEFs with non-targeting or nesprin-2 siRNA and subjected them to subcellular fractionation by differential centrifugation. We then monitored the expression of Bcl-x_L_, Bak, and Bax in cytosolic and heavy membrane (HM) fractions by immunoblotting. The HM fraction is enriched with mitochondria as indicated by the presence of the mitochondrial marker Tom20 (Fig. 7A). As expected, in cells treated with non-targeting siRNA, Bak was mainly mitochondrial (~85%), whereas the portion of mitochondrial Bax and Bcl-x_L_ was lower (~20%) (Fig. 7A and B). Treatment of these cells with cisplatin significantly increased the portion of mitochondrial Bcl-x_L_, but not Bax (Fig. 7A and B). Nesprin-2 depletion significantly increased the mitochondrial portion of Bax in both cisplatin-untreated and treated cells. Nesprin-2 depletion also significantly increased the mitochondrial portion of Bcl-x_L_, but only in cisplatin-untreated cells. This is probably because nesprin-2 depletion could not further increase the mitochondrial portion of Bcl-x_L_ beyond that increased by cisplatin. Similarly, nesprin-2 depletion did not affect the high mitochondrial portion of Bak (Fig. 7A and B). These results suggest that nesprin-2 depletion regulates mitochondrial translocation/retrotranslocation of Bcl-x_L_ and Bax.

Next, we assessed the effect of non-targeting or nesprin-2 siRNA on the mitochondrial portion of Bax and Bak in Bcl-x_L_ KO cells. As shown in WT MEFs, nesprin-2 depletion in Bcl-x_L_ MEFs did not affect mitochondrial portion of Bak. However, in contrast to WT MEFs, where nesprin-2 depletion increased the portion of mitochondrial Bax, nesprin-2 depletion in Bcl-x_L_ MEFs did not change the mitochondrial portion of Bax (Fig. 7C and D). Taken together, these results suggest that nesprin-2 depletion regulates mitochondrial translocation/retrotranslocation of Bcl-x_L_ and Bax, and that Bcl-x_L_ is needed for the regulation of mitochondrial Bax translocation/retrotranslocation by nesprin-2 depletion.

## Discussion

Nesprin-2 is a component of the LINC complex that connects the cytoskeleton to the NE. We show that nesprin-2 has also pro-apoptotic activity. Accordingly, nesprin-2 depletion by siRNA and shRNA that most likely depletes all isoforms, reduced cell death and inhibited key events in the mitochondrial apoptotic pathway, namely Bax and Bak activation and MOMP. These findings suggest that nesprin-2 can act as an apoptotic regulator. The pro-apoptotic action of nesprin-2 can be mediated via its effect on Bcl-2 family proteins, namely by regulating their mitochondrial translocation/retrotranslocation, by inhibiting the action of the pro-survival protein Bcl-x_L_ and by activating the pro-apoptotic protein Bak independently of Bcl-x_L_. These nesprin-2 actions ultimately promote MOMP and apoptotic cell death.

Alternative RNA splicing for *Syne2* gene transcripts can generate multiple nesprin-2 isoforms, of which the largest is nesprin-2G, which is known to interact with both actin and microtubules. Shorter isoforms lacking the actin-binding CH domains interact with microtubules. In addition, KASH-less isoforms have also been reported [5, 36]. Our results show that depletion of nesprin-2G alone does not inhibit apoptosis. Whether the nesprin-2 pro-apoptotic function is mediated by other nesprin-2 isoforms, or by a combined action of several isoforms including nesprin-2G is currently unknown and needs further investigation. Nonetheless, our previous results showing that in apoptotic cells nesprin-2G binds Bax in close proximity to the mitochondria [26] and our current results showing that nesprin-2G siRNA inhibits Bak-NT exposure in Bcl-x_L_ KO cells indicate that it participates in the overall nesprin-2 apoptotic effect.

We showed previously that during apoptosis nuclear proteins are redistributed from the nucleus [24] and that nesprin-2 redistributes from the NE [26]. These results may imply that the pro-apoptotic role of nesprin-2 is mediated by the redistributed nuclear proteins or the redistributed nesprin-2. However, our current results showing that nesprin-2 depletion does not inhibit NPR suggest that the survival effect of its depletion is not mediated via NPR inhibition. Nesprin-2 is also not needed for apoptosis-induced NPR. Redistribution of nesprin-2 from the NE by GFP-KASH neither increased nor decreased sensitivity to apoptosis. This indicates that the absence of nesprin-2 from the NE per se does not inhibit apoptosis and its redistribution out of the NE does not contribute to cell death. However, we cannot exclude the possibility that the nature (e.g., cleavage, post-translation modification, etc.) of nesprin-2 redistributed in response to apoptotic triggers differs from the nesprin-2 that is displaced from the NE by GFP-KASH and thus that nesprin-2 redistributed during apoptosis may play a role in the pro-apoptotic action of nesprin-2.

### Nesprin-2 may inhibit Bcl-x_L_ activity and regulate mitochondrial translocation/retrotranslocation of Bcl-2 family proteins

Depletion of nesprin-2 did not promote cell survival in the absence of Bcl-x_L_. This suggests that the pro-apoptotic effect of nesprin-2 is mediated by inhibiting the anti-apoptotic action of Bcl-x_L_. This conclusion is further supported by our findings that depleting nesprin-2 did not inhibit apoptosis induced by ABT-373, which acts by inhibiting Bcl-x_L_ activity. The mechanism whereby nesprin-2 inhibits the action of Bcl-x_L_ is currently unknown. Potential mechanisms may involve binding of nesprin-2 to Bcl-x_L_ or other anti-apoptotic Bcl-2 family proteins e.g., Bcl-2, which is also inhibited by ABT-373 [37]. Such binding will prevent Bcl-x_L_ from inhibiting Bax and/or Bak, which in turn will induce Bax/Bak activation and generation MOMP pores and/or megaspores [38]. The binding of nesprin-2 to Bcl-x_L_ could also affect the mitochondrial translocation/retrotranslocation of Bcl-x_L_ and Bax, a process known to occur through interactions between Bax and anti-apoptotic Bcl-2 family proteins [15]. In that regard, our results show that nesprin-2 depletion increases the mitochondrial portion of Bcl-x_L_ and Bax and that the effect of nesprin-2 depletion on the mitochondrial portion of Bax was abolished in Bcl-x_L_ KO MEFs. Nesprin-2 depletion did not affect the mitochondrial portion of Bak, probably because of its low shuttling rate which causes predominant mitochondrial Bak localization [34, 39]. We cannot exclude the possibility that other mechanisms may regulate the effect of nesprin-2 on translocation/retrotranslocation of Bcl-2 family members, such as an effect on mitochondria positioning [40] or on cytoskeleton organization. Regarding the latter, retromer, a conserved endosomal scaffold complex involved in membrane trafficking, facilitates the localization of Bcl-x_L_ to the mitochondria [41]. Since nesprin-2 can interact with microtubule motors [11] that facilitate intracellular trafficking, it may regulate the endosomal membrane trafficking-mediated delivery of Bcl-2 family proteins to the mitochondria. Additional studies are needed to unravel the mechanism whereby nesprin-2 regulates the mitochondrial translocation/retrotranslocation of Bcl-2 family members.

### Nesprin-2 may directly induce Bak activation

Our results show that in healthy MEFs lacking Bcl-x_L_ mitochondrial Bak exhibits an activated conformation of NT exposure (detected by Bak-NT Abs). This mitochondrial Bak-NT exposure probably occurs by spontaneous Bak activation in the mitochondria because of the lack of Bcl-x_L_. The reason that these Bcl-x_L_ lacking MEFs do not die, albeit this initial step in Bak activation, is probably due to the protective effects of other anti-apoptotic Bcl-2 family members, which prevents complete Bak activation. Our finding that nesprin-2 depletion inhibited this Bak-NT exposure in cells lacking Bcl-x_L_, suggests that nesprin-2 can promote Bak activation by Bcl-x_L_-independent manner. This result further implies that nesprin-2 can contribute to apoptosis also independently of its inhibitory effect on Bcl-x_L_, perhaps by directly activating Bak. An alternative explanation could be that nesprin-2 depletion shifts the equilibrium of mitochondrial Bak translocation/retrotranslocation [34] towards retrotranslocation from the mitochondria, which in turn prevents the appearance of mitochondrial Bak NT. However, the results showing that nesprin-2 depletion does not affect the portion of mitochondrial Bak argue against this possibility.

Notably, nesprin-2G depletion also inhibited Bak-NT exposure in Bcl-x_L_ KO cells, although to a lesser extent than depletion of all nesprin-2 isoforms. This supports the notion that nesprin-2G participates in the pro-apoptotic action of nesprin-2. The effect of nesprin-2 on Bak-NT exposure or on other Bcl-2 family proteins in mitochondria may require their localization in close proximity to the mitochondria. Consistent with this notion, members of the nesprin family, including nesprin-2, have been shown by others to localize to the mitochondria [6, 40, 42] and we have also previously shown that during apoptosis nesprin-2 redistributes from the NE to regions close to mitochondria [26]. Although nesprin-2 depletion inhibited spontaneous Bak-NT exposure in Bcl-x_L_ KO MEFs, it did not inhibit Bak activation in apoptotic Bcl-x_L_ KO MEFs. This was likely because full Bak activation is caused by the action of Bcl-x_L_-inhibitable BH3-only proteins. Thus, in the absence of Bcl-x_L_, nesprin-2 depletion cannot execute its Bcl-x_L_-dependent inhibitory effect on Bak activation.

## Conclusion

**Fig. 8:**
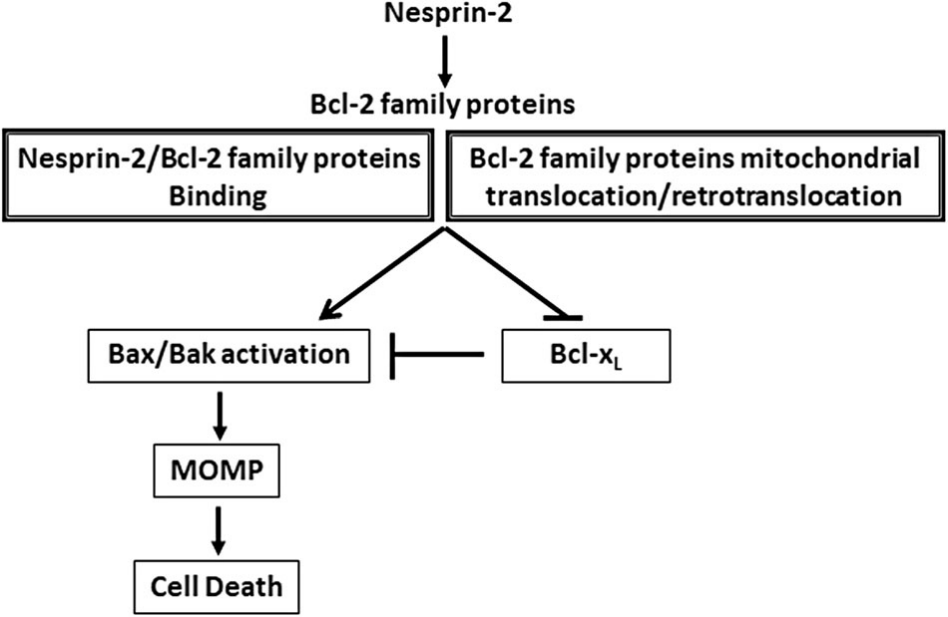
Model illustrating the proposed role of nesprin-2 in regulating apoptosis. Nesprin-2 regulates apoptosis via its effect on both anti- and pro-apoptotic Bcl-2 family proteins. This regulation can be mediated by binding to anti- and pro-apoptotic Bcl-2 family proteins and/or by controlling the mitochondrial translocation/retrotranslocation of these proteins. These nesprin-2 effects will lead to inhibition of the anti-apoptotic activity of Bcl-x_L_ which in turn will promote Bax/Bak activation as well as directly induce Bak activation. Ultimately, these effects will culminate in MOMP and apoptotic cell death.

We have shown that nesprin-2 can act as a pro-apoptotic protein that regulates the mitochondrial apoptotic pathway. We propose (Fig. 8) that the nesprin-2 apoptotic effect is mediated via its action on Bcl-2 family proteins by inhibiting the anti-apoptotic effect of Bcl-x_L_, by directly inducing Bak activation as well as by regulating the translocation/retrotranslocation of both anti- and pro-apoptotic Bcl-2 family members to and from the mitochondria, all may lead to MOMP. These effects may involve a direct interaction between nesprin-2 and Bcl-2 family proteins. This notion is supported by our previous findings which showed that nesprin-2 can bind Bax in close proximity to the mitochondria [26]. Nesprin-2 binds to several proteins including actin, actin-binding and regulatory proteins [8, 10, 43] and the dynein/kinesin “adapter” BicD2 [11]. Thus, nesprin-2 may serve as a scaffold protein that also binds anti- and pro-apoptotic Bcl-2 family proteins. Such binding may directly promote Bax/Bak activation or inhibit Bcl-x_L_ activity by sequestering Bcl-x_L_ or by inhibiting the ability of Bcl-x_L_ to neutralize the pro-apoptotic Bcl-2 proteins. Alternatively, it may play a role in the nesprin-2-regulated mitochondrial translocation/retrotranslocation of Bcl-2 family proteins. Further studies are needed to unravel the interplay between nesprin-2 and the different Bcl-2 family members. In summary, our findings identify a new function for the NE protein nesprin-2 as an apoptotic regulator. They further support the notion that the NE is not just a target of the apoptotic machinery, but may also act as an apoptotic mediator [12].

## Materials and methods

### Materials

All reagents were purchased from Sigma-Aldrich unless otherwise specified. Quinoline-VaL-Asp(OMe)-CH2-OPH (Q-VD-OPH) was purchased from Apex Biotechnology. ABT-737 (Cat#HY-50907) was purchased from MedChemExpress.

### Cell culture

Immortalized wild type (WT) 3T9 MEFs [23] and *Bcl2l1*^*−/−*^/*Bcl-x*^*−/−*^ (herein *Bcl-x*^*−/−*^), kindly provided by Prof. Christoph Borner (University of Freiburg, Germany), were grown in Dulbecco’s modified Eagle’s medium containing 10% fetal bovine serum at 37°C and 5% CO_2_. The cells were routinely checked for mycoplasma.

### Plasmids

The generation of GFP-KASH expression vector has been described previously [44]. The vector used to specifically target the region of the *Syne2* gene encoding nesprin-2G was generated by inserting an oligonucleotide of sequence 5′-TTACAATCAACCTTCACCGG-3′ into the BbsI site in of pSpCas9(BB)-2A-GFP (PX458) (Addgene) to generate pSpCas9(BB)-2A-GFP-nesprin-2G.

### Antibodies

The following antibodies (Abs) were used for immunofluorescence (IF) microscopy and immunoblotting: mouse anti-Bax Clone 6A7 (#556467) (BD Biosciences) [dilution 1:50 for IF staining], mouse anti-cytochrome-*c* (#556432) (BD Biosciences) (dilution 1:200 for IF staining), mouse anti-nucleophosmin (anti-B23 #B0556) (Sigma-Aldrich) (dilution 1:200 for IF staining), rabbit anti-Bax N-terminus (NT) (#ABC11) (Millipore, Merck) (dilution 1:1000 for immunoblotting), rabbit anti-Bak NT (#06-536) (Millipore, Merck) (dilution 1:1000 for immunoblotting and 1:100 for IF staining), rabbit anti-Bcl-x_L_ (#2762) (Cell Signaling Technology) (dilution 1:1000 for immunoblotting) and 1:200 for IF staining, rat anti-Bcl-w (#MC-501) (Kamiya Biomedical Company) (dilution 1:1000 for immunoblotting), goat anti-Bid (#AF860) (R&D Systems) (dilution 1:10,000 for immunoblotting), rabbit anti-Mcl-1 (#600-401-394) (Rockland) (dilution 1:1000 for immunoblotting), mouse monoclonal anti-lamin A/C (#SAB4200236) (Sigma-Aldrich) (dilution 1:6000 for immunoblotting), rabbit anti-Tom20 (#42406) (Cell Signaling Technology) (dilution 1:1000 for immunoblotting), mouse anti-tubulin (#T4026) (Sigma-Aldrich) (dilution 1:2500 for immunoblotting), rabbit anti-nesprin-2G (dilution 1:200 for IF staining) [29], rabbit anti-nesprin-2 K2 [45], rabbit anti-nesprin-1 [45], rabbit anti-nesprin-3 [46] and rabbit anti-Sun2 (#Ab87036) (Abcam) (dilution 1:1000 for immunoblotting).

### Transfection

Transfections were performed using the TransIT-X2 transfection Reagent (Mirus Bio LLC) according to the manufacturer’s instructions.

### siRNA-mediated knockdown

For nesprin-2 knockdown, WT or *Bcl-x*^*−/−*^ MEFs were transfected with Dharmacon SMART pool ON-TARGETplus mouse *Syne2* siRNA (Cat#L-056764-02) designed to target regions within nesprin-2 exons 92-93, 95, 102 and 103. For knockdown of nesprin-2G, WT MEFs were transfected with custom Dharmacon ON-TARGETplus siRNA designed to target a region within the end of exon 8 and beginning of exon 9 of mouse *Syne2* (5′ -AACCAGAAGAUGUAGAUGUUU- 3′) according to [47]. For Bcl-x_L_ knockdown, WT MEFs were transfected with Dharmacon ON-TARGETplus SMARTpool mouse *Bcl2l1* siRNA (L-065142-00). For control non-targeting siRNA, WT MEFs were transfected with Dharmacon ON-TARGETplus Non-targeting Pool siRNA (D-001810-10). For knockdown of nesprin-2 or nesprin-2G in WT and *Bcl-x*_*L*_^*−/−*^ MEFs, 50 nM of nesprin-2 or nesprin-2G siRNAs oligonucleotides were used. For the combined knockdown of nesprin-2 and bcl-x_L_ in WT MEFs, 25 nM of nesprin-2 and 50 nM of bcl-x_L_ siRNAs oligonucleotides were used. In the corresponding controls, nesprin-2 or bcl-x_L_ were separately knocked down using 25 nM or 50 nM of nesprin-2 or bcl-x_L_ oligonucleotides respectively, together with 50 nM or 25 nM non-targeting oligonucleotides respectively. Cells were transfected with the different siRNAs and three days later were incubated for an additional 24 h in media with 25 µM cisplatin, 100 nM staurosporine, 10 µM ABT-737 plus 1 µM actinomycin D with 20 µM Q-VD-OPH or no drug.

### Stable knockdown of nesprin-2 in WT MEFs

WT MEFs were infected with pSUPER retro puro virus (Oligoengine) encoding mouse nesprin-2 shRNA [27] [mouse nesprin-2 shRNA targeting the sequence in the 3′ UTR (5′-GCACGTAAATGACCTATAT-3′)] or with pSUPER retro puro virus encoding scramble (negative control) shRNA (5′-CAACAAGTAGAAGAGAGCACCAA-3′). Viruses were produced in HEK293T cells. Viruses were added to subconfluent cultures of WT MEFs in the presence of 2 μg/ml polybrene (Millipore, Billerica, MA) and cells were re-plated at low density on the next day in the presence of 1 µg/ml puromycin. Puromycin-resistant nesprin-2 shRNA clones or negative control pool were isolated and assessed for nesprin-2 expression.

### Generation of Syne2 knockout MEF clones

WT MEFs were transfected with pSpCas9(BB)-2A-GFP-nesprin-2G vector to promote CRISPR/Cas9 editing of nesprin-2G isoform. Three days after transfection cells were re-plated at single cell density and further grown to obtain subclones. Subclones were then isolated expanded and tested for nesprin-2G expression by immunoblotting. Subclones that lack nesprin-2G expression were used in the subsequent experiments.

### IF microscopy

10^5^ cells were seeded and grown on 18-mm cover slips coated with collagen. After treatments, cells were fixed and stained with the indicated antibodies together with Hoechst 33258 dye, as described previously [48]. Cells were co-stained with anti-nesprin-2G and anti-Bax 6A7 or with anti-cytochrome *c* and anti-Bak NT Abs. Images were captured using a fluorescence microscope (EVOS Cell Imaging Systems, Thermo Fisher Scientific) or confocal microscope (LEICA TCS SP5 II) using Zeiss X63 NA 1.4 objective lens.

Cells exhibiting the indicated apoptotic events were counted under the fluorescence microscope. In the case of Bak-NT staining in healthy *Bcl-x*_*L*_^*−/−*^ MEFs or WT MEFs treated with Bcl-x_L_ and nesprin-2 siRNAs, 15 random images from each treatment group were captured using confocal microscope, and the number of cells in the different treatments exhibiting Bak-NT staining pattern of elongated structures were blindly counted.

### Immunoblotting

Total cell lysates were prepared and analyzed by immunoblotting as previously described [26]. Proteins were separated on 4–15% Criterion TGX Precast Gradient Gel (Bio-Rad Laboratories) or 12.5% SDS-PAGE and electroblotted (3 h, 80 V, in the case of the 4-15% gels or 1 h, 100 V in the case of 12.5% SDS-PAGE) onto nitrocellulose membranes. The resulting blots were probed with LINC complex Abs or Bcl-2 family proteins Abs respectively. In the nesprin-2 shRNA experiment, proteins were separated on 10% SDS-PAGE, electroblotted (1 h, 100 V) onto nitrocellulose membranes, and probed with anti-nesprin-2 Ab. Uniformity of sample loading was verified based on the Ponceau staining of the blots as internal control. Normalization of protein expression level was performed relative to bands detected by the Ponceau staining. Accordingly, the signal intensity values of immunoblots bands were normalized to the intensity values of selected proteins bands detected by the Ponceau staining; a 15 KDa band, when proteins were electroblotted for 3 h, or 30–45 KDa bands when proteins were electroblotted for 1 h.

### Assessments of apoptotic events

Apoptotic events were identified in IF micrographs by cells exhibiting (i) cytochrome *c* release, defined by appearance of a diffused staining pattern of cytochrome *c*. (ii) Bax or Bak activation defined by their NT exposure detected using anti-Bax 6A7 and anti-Bak NT Abs, respectively.

### Subcellular fractionation

WT (5 × 10^5^/6 cm dish) or *Bcl-x*_*L*_^*−/−*^ (1.5 × 10^6^/10 cm dish) MEFs were transfected with non-targeting or nesprin-2 siRNA oligonucleotides. Three days later, cells were harvested, resuspended in 0.5 ml of mitochondrial homogenization buffer containing 200 mM mannitol, 70 mM sucrose, 1 mM EGTA, 10 mM HEPES (pH 7.5), and protease inhibitor cocktail (Chalbiochem #539131), incubated for 1 h on ice and then homogenized with a Dounce homogenizer (60 strokes). Samples were checked microscopically to ensure that most cells were lysed and centrifuged at 500 *g* for 5 min at 4°C to pellet the nuclei. The post-nuclear supernatant was then centrifuged at 13,000 *g* for 10 min at 4°C. The resulting pellet, representing heavy membranes, was resuspended with equivalent volume of the supernatant with ice-cold homogenization buffer. The supernatant of the final centrifugation represents the cytosolic fraction. Equivalent proportions of each fraction (heavy membranes and cytosol) were subjected to SDS-PAGE (12.5%) immunoblot analysis using the indicated antibodies.

### Statistical analysis

The sample size for the experiments were chosen in advance according to previous experiments using the same assays [24, 26]. Statistical significance was determined using one or two-way ANOVA followed by Dunnett’s test or Student’s *t*-test. For the statistical analysis of Fig. 7B, after two-way ANOVA, the results were further assessed by simple main effects test (using linear contrasts) with correction for multiple comparisons using the False Discovery Rate method (two-stage FDR method of Benjamini, Krieger, and Yekutieli) [49]. The experiment of activated Bak (Fig. 6G), was run at two different time periods, thus it was analyzed using a two-way ANOVA, where the first factor represents the treatment and the second factor represents time, followed by Dunnett’s test. All data sets were validated for meeting all assumptions of the tests (e.g., normal distribution and equality of variances). Values of *p* < 0.05 or *q* < 0.05 were considered statistically significant. Data are expressed as mean values ± SEM.

### Supplementary information

Supplementary Information

Original Data File

## Data Availability

All the data is available in the main text or in supplementary material as stated in the text. The full length uncropped original western blots are provided in the Supplementary Material file.
