# Supplementary material for: LINC complex protein nesprin-2 has pro-apoptotic activity via Bcl-2 family proteins
Source: Cell Death Discov. 2024 Jan 15;10:29. doi: 10.1038/s41420-023-01763-w (PMC10789774; doi:10.1038/s41420-023-01763-w)
Supplement: Supplementary file 1 — Supplementary Information [file 41420_2023_1763_MOESM1_ESM.pdf]

LINC complex protein nesprin-2 has pro-apoptotic activity via Bcl-2 family proteins

Cell Death and Discovery

Liora Lindenboim[1], Hila Zohar[1], Gregg G Gundersen[2], Howard J Worman[2,3] and Reuven Stein[1]*

[1]Department of Neurobiology, School of Neurobiology, Biochemistry and Biophysics, George S. Wise Faculty of Life Sciences, Tel Aviv University, Ramat Aviv 69978, Israel

[2]Department of Pathology and Cell Biology, Vagelos College of Physicians and Surgeons, Columbia University, New York, NY 10032, USA.

[3]Department of Medicine, Vagelos College of Physicians and Surgeons, Columbia University, New York, NY 10032, USA.

*Corresponding author: R. Stein;

E-mail: reuvens@tauex.tau.ac.il

Figure S1

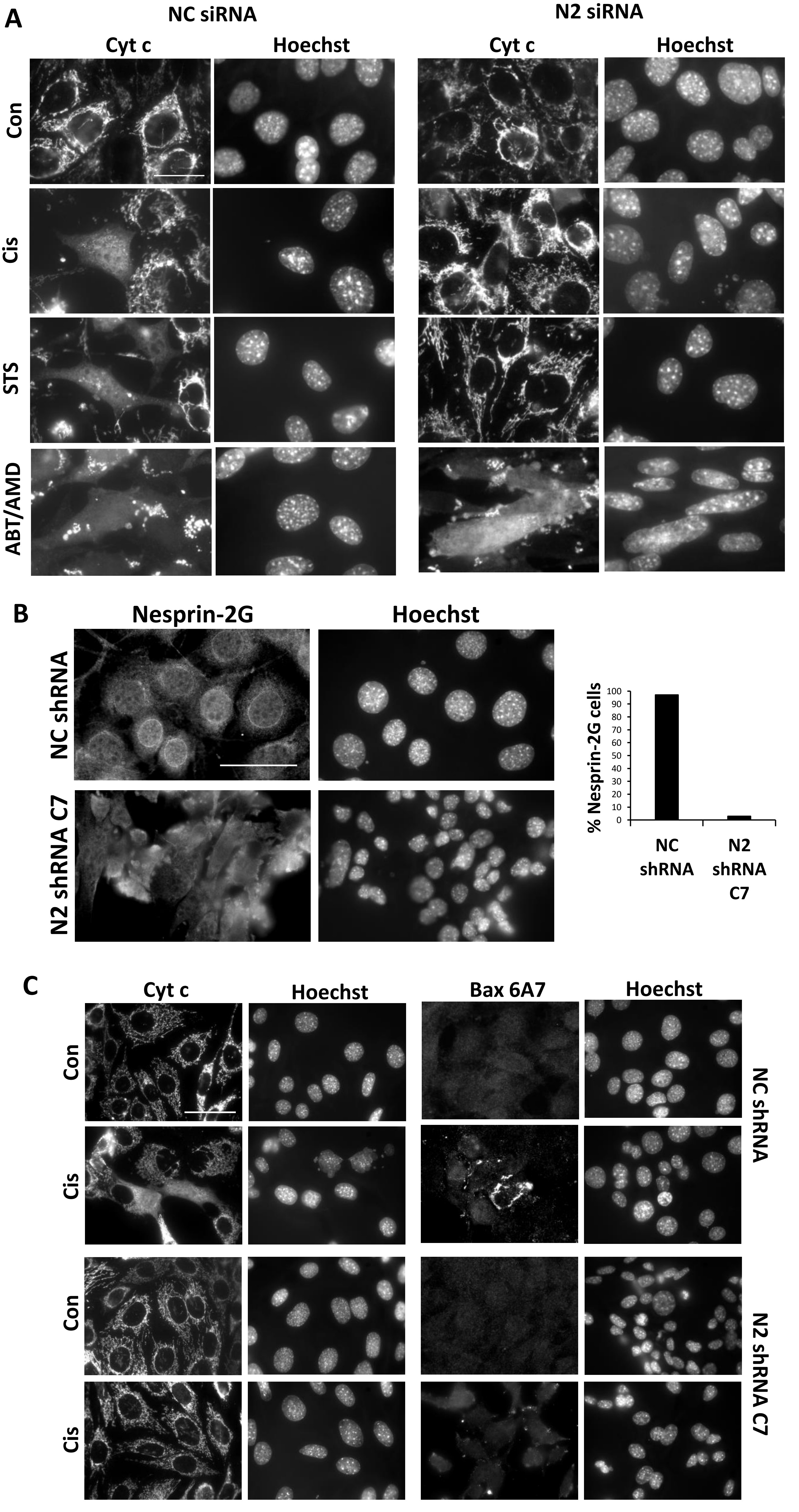
A
NC siRNA
N2 siRNA
Cyt c
Hoechst
Cyt c
Hoechst
Con
Cis
STS
ABT/AMD
B
Nesprin-2G
Hoechst
NC shRNA
N2 shRNA C7
% Nesprin-2G cells
100
90
80
70
60
50
40
30
20
10
0
NC shRNA
N2 shRNA C7
C
Cyt c
Hoechst
Bax 6A7
Hoechst
Con
Cis
Con
Cis
NC shRNA
N2 shRNA C7

Fig. S1 The effect of nesprin-2 depletion on apoptotic parameters. (A) Effect of nesprin-2 siRNA on cytochrome *c* release. WT MEFs were treated as described in Fig. 1C. The results show representative fluorescence microscopy micrographs of cytochrome *c* (Cyt c) staining in WT MEFs, untreated (Con) or treated with cisplatin (Cis), staurosporine (STS) or ABT-737 and actinomycin D (ABT/AMD) together with Q-VD-OPH. The images shown for cisplatin-treated WT MEFs are shown for comparison. Bar = 25 µm (*n* = 5). (B, C) Effects of nesprin-2 shRNA in negative control (NC shRNA) and nesprin-2 shRNA clone 7 (N2 shRNA C7). (B) Effect on nesprin-2G expression. The left panel shows representative IF micrographs of N2 shRNA C7 cells stained with anti-nesprin-2G Ab and Hoechst dye. Images were captured by fluorescence microscopy. The images show the same field visualized separately for detecting nesprin-2G and nuclei staining. Bar = 50 µm. The right panel shows quantification of the percentage of cells exhibiting NE-associated nesprin-2G. (C) The effect of nesprin-2 shRNA on cisplatin-induced cytochrome *c* release and Bax-NT exposure in the N2 shRNA C7 cells, untreated (Con) or treated with cisplatin and Q-VD-OPH (Cis). The cells were stained with Hoechst dye and with anti-cytochrome *c* or anti-Bax 6A7 Ab. The images shown are representative images (*n* = 3).

Figure S2

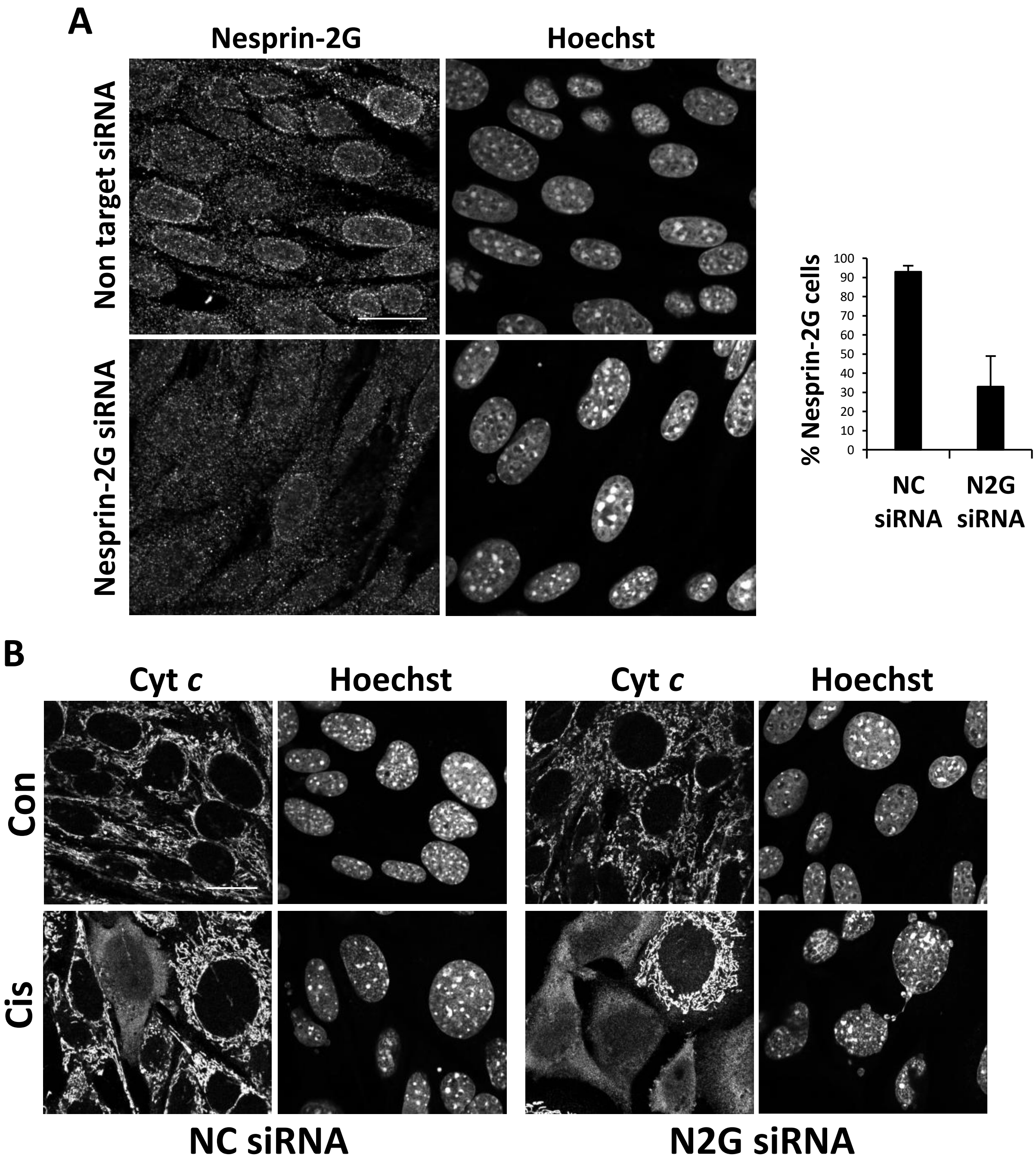
A
Nesprin-2G
Hoechst
Non target siRNA
Nesprin-2G siRNA
% Nesprin-2G cells
100
90
80
70
60
50
40
30
20
10
0
NC siRNA
N2G siRNA
B
Cyt c
Hoechst
Cyt c
Hoechst
Con
Cis
NC siRNA
N2G siRNA

Figure S2

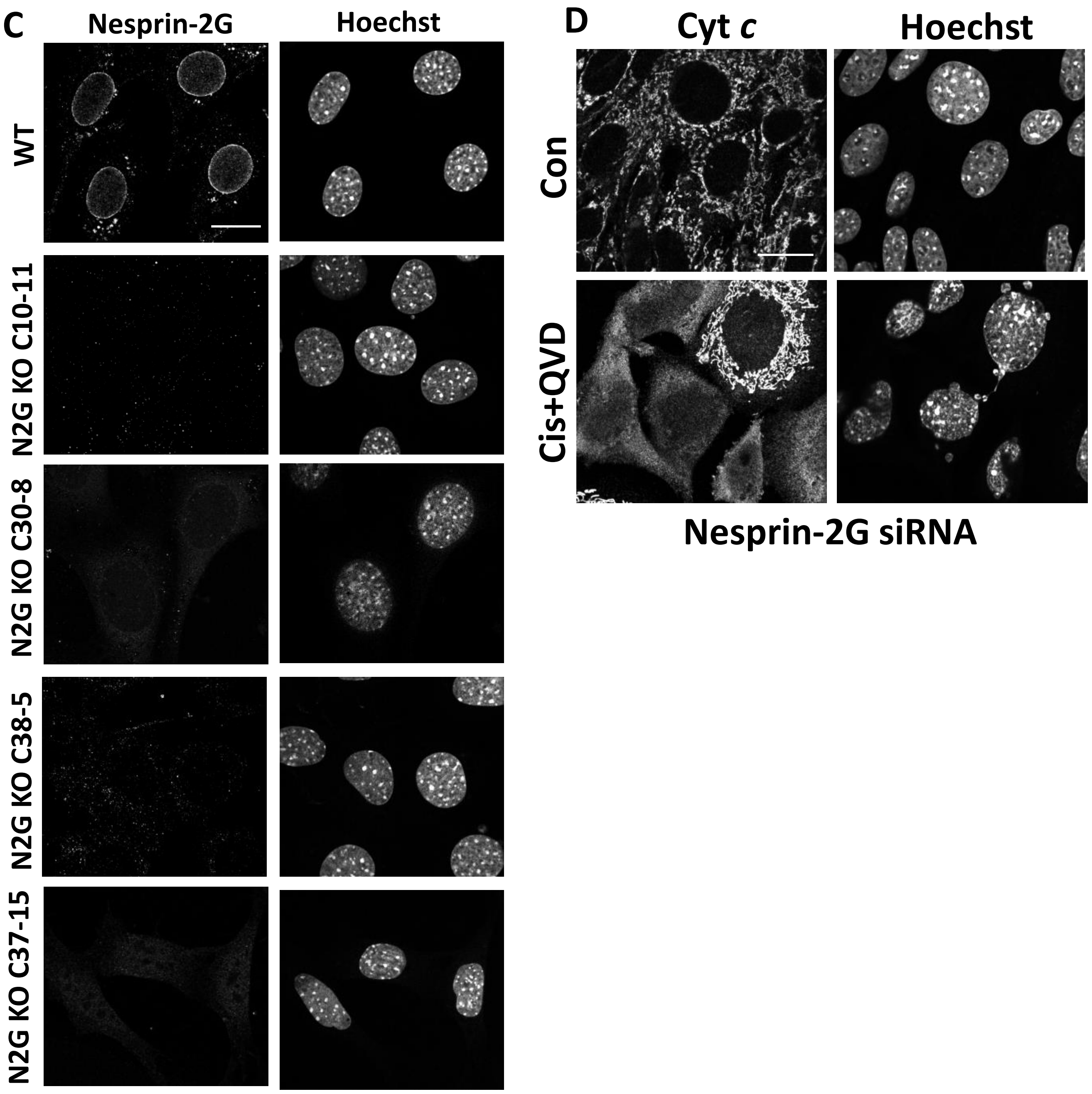

Fig. S2 Nesprin-2G depletion does not affect cisplatin-induced cytochrome *c* release. (A, B) Nesprin-2G siRNA downregulates nesprin-2G expression but does not affect cisplatin-induced cytochrome *c* release. WT MEFs were transfected with non-targeting [negative control (NC)] siRNA (NC siRNA) or nesprin-2G (N2G) siRNAs. (A) The left panel shows representative micrographs of IF staining of nesprin-2G in the indicated siRNAs transfected cells stained with nesprin-2G Ab and Hoechst dye, 72 h after transfection. The right panel shows quantification of the percentage of cells exhibiting NE-associated nesprin-2G (n = 3).
(B) Representative micrograph of cytochrome *c* (Cyt c) IF staining of the indicated siRNAs transfected cells, untreated (Con) or treated with cisplatin and Q-VD-OPH for 24 h. (C, D) Nesprin-2G knockout clones. (C) IF micrographs of nesprin-2G expression in WT MEFs and nesprin-2G (N2G) knockout (KO) clones C10-11, C30-8, C38-5 and C37-15. (D) The effect of cisplatin (Cis) treatment on cytochrome *c* release in N2G KO C37-15 clone. Cells were treated with cisplatin and Q-VD-OPH for 24 h and analyzed for cytochrome *c* release. The images shown are representative images (n = 3).

Figure S3

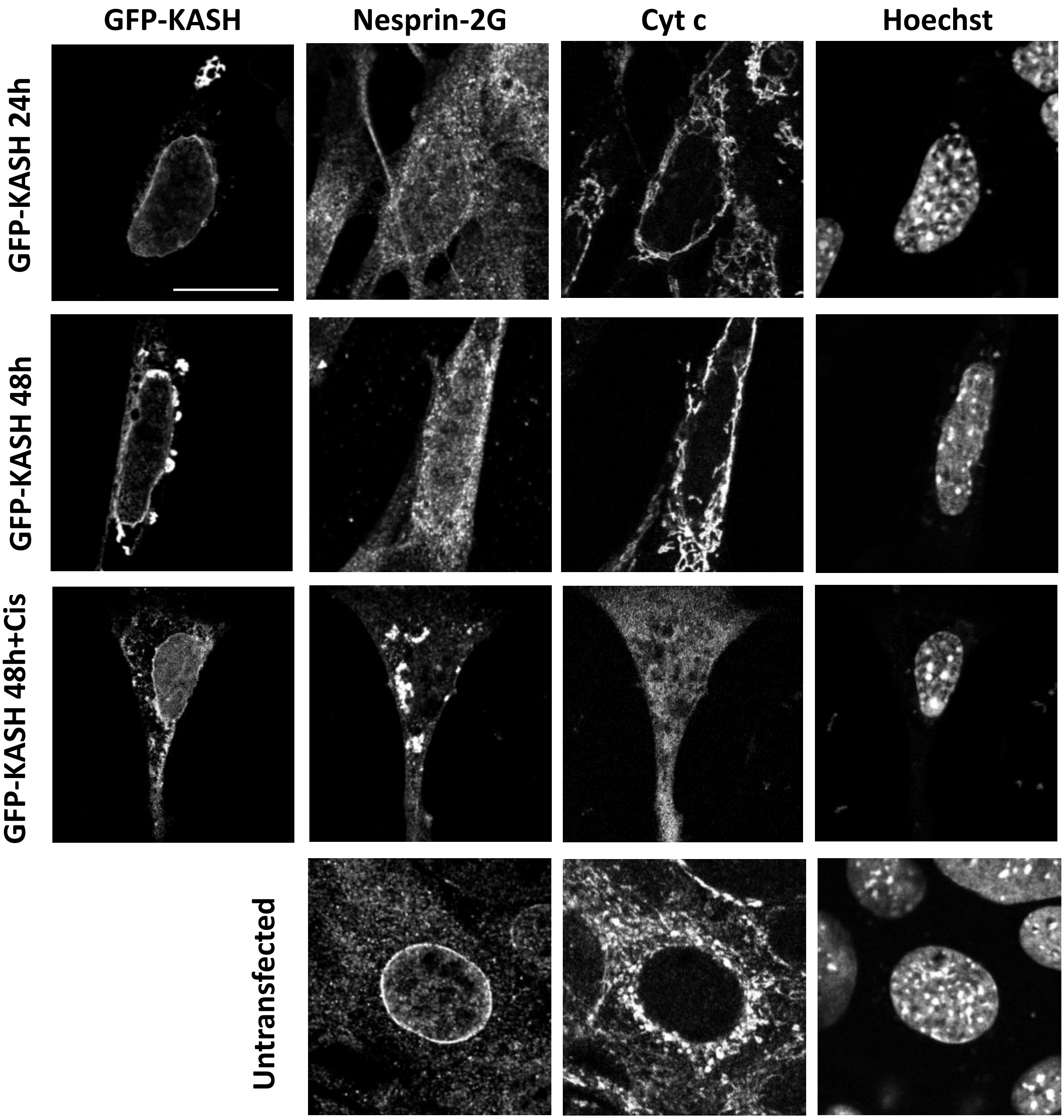

Fig. S3 GFP-KASH expression induces displacement of nesprin-2 from the NE, but not cytochrome *c* release. WT MEFs untransfected or transfected with GFP-KASH expression vector were treated as described in Fig. 4. The results shown are representative IF micrographs of cells stained with Hoechst and anti-cytochrome *c* (Cyt *c*) or anti-nesprin-2G Abs and visualized by confocal fluorescence microscopy (*n* = 3).

Figure S4

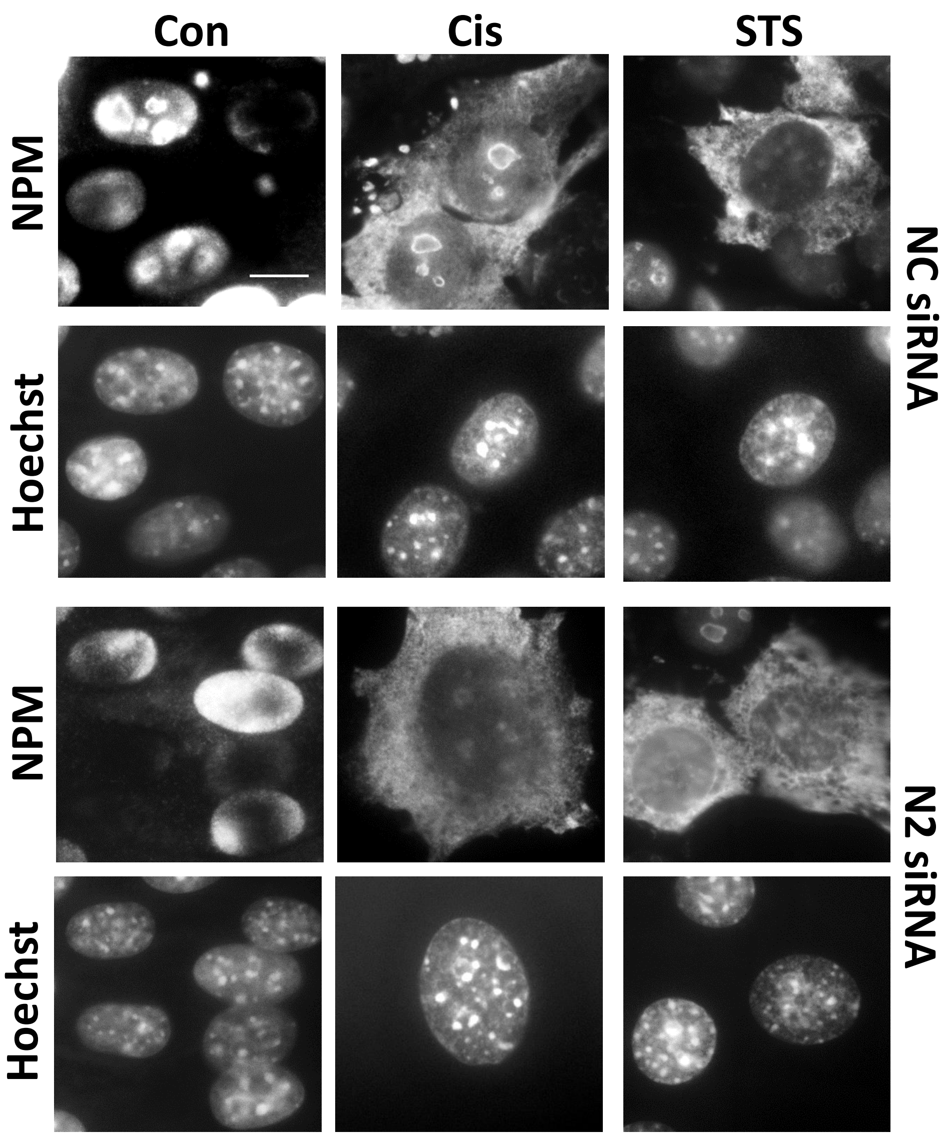

Fig. S4 Nesprin-2 depletion does not affect nucleophosmin redistribution. Representative IF micrographs of nucleophosmin (NPM) staining in cells treated with no drug (Con) or with cisplatin (Cis) and Q-VD-OPH as described in Fig. 5. WT MEFs were transfected with non-targeting [negative control (NC)] or nesprin-2 (N2) siRNAs and untreated or treated with cisplatin and Q-VD-OPH as described in Fig. 5. Then, the cells were IF stained with anti-nucleophosmin (NPM) Ab and visualized by fluorescence microscopy (*n* = 3). Bar = 10 µm

Figure S5

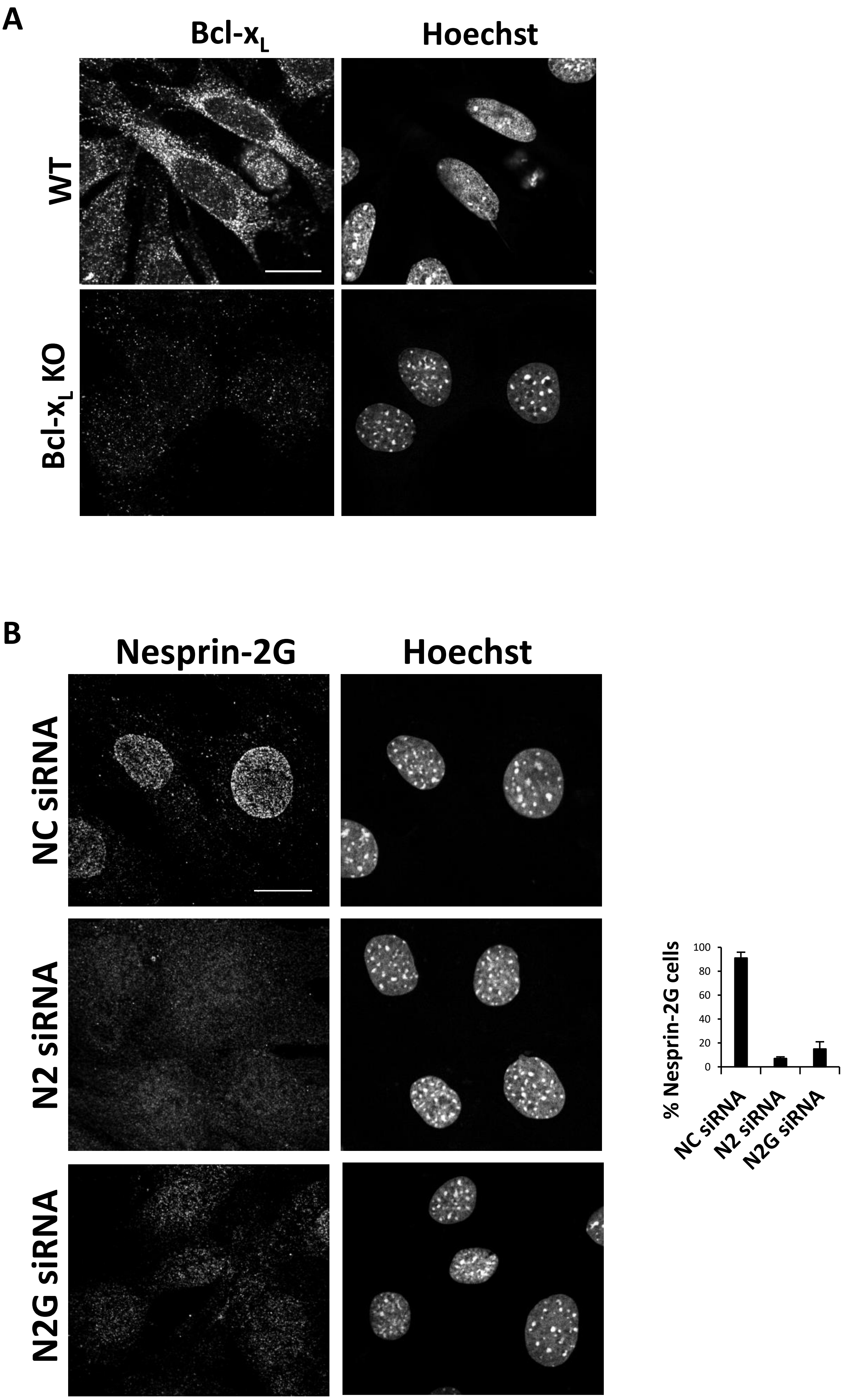
A
Bcl-xL
Hoechst
WT
Bcl-xL KO
B
Nesprin-2G
Hoechst
NC siRNA
N2 siRNA
N2G siRNA
% Nesprin-2G cells
100
80
60
40
20
0
NC siRNA
N2 siRNA
N2G siRNA

Figure S5

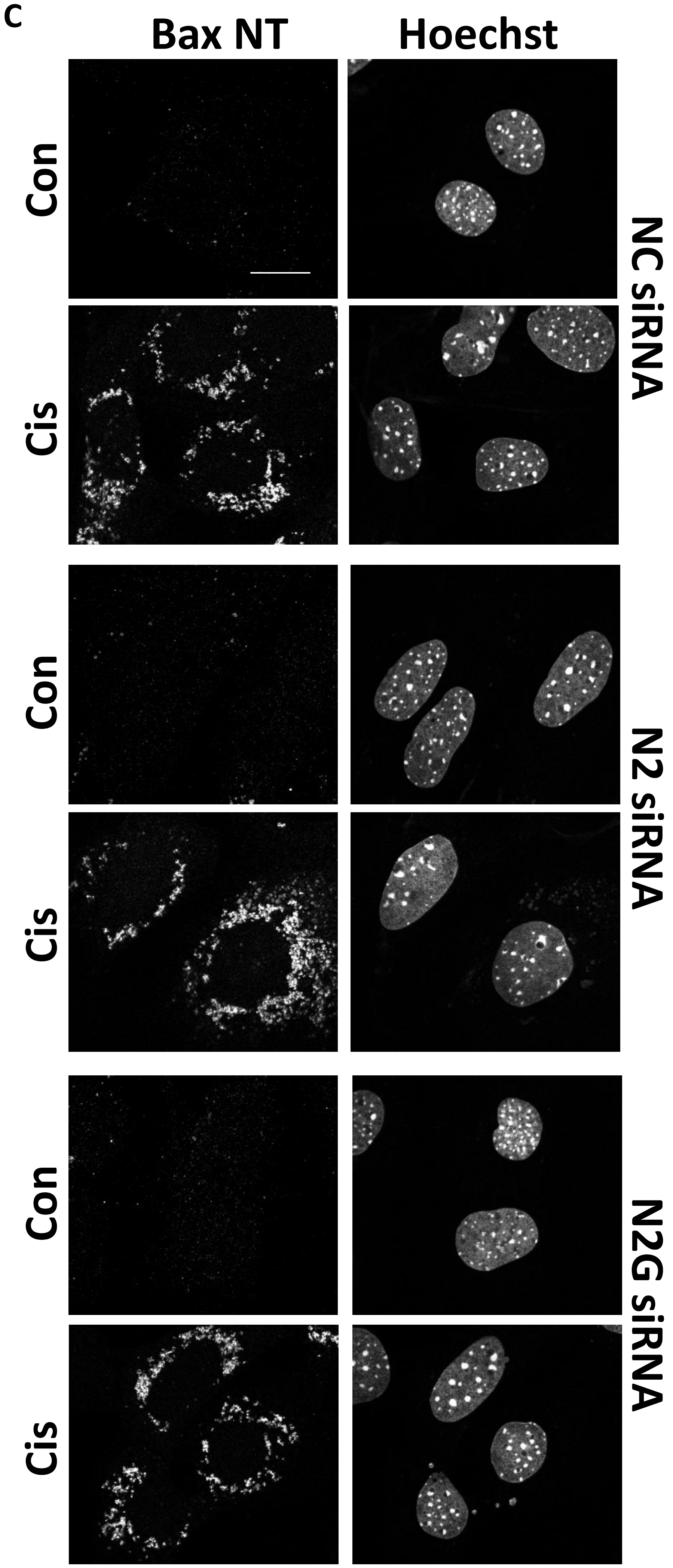
C
Bax NT
Hoechst
Con
Cis
NC siRNA
Con
Cis
N2 siRNA
Con
Cis
N2G siRNA

Figure S5

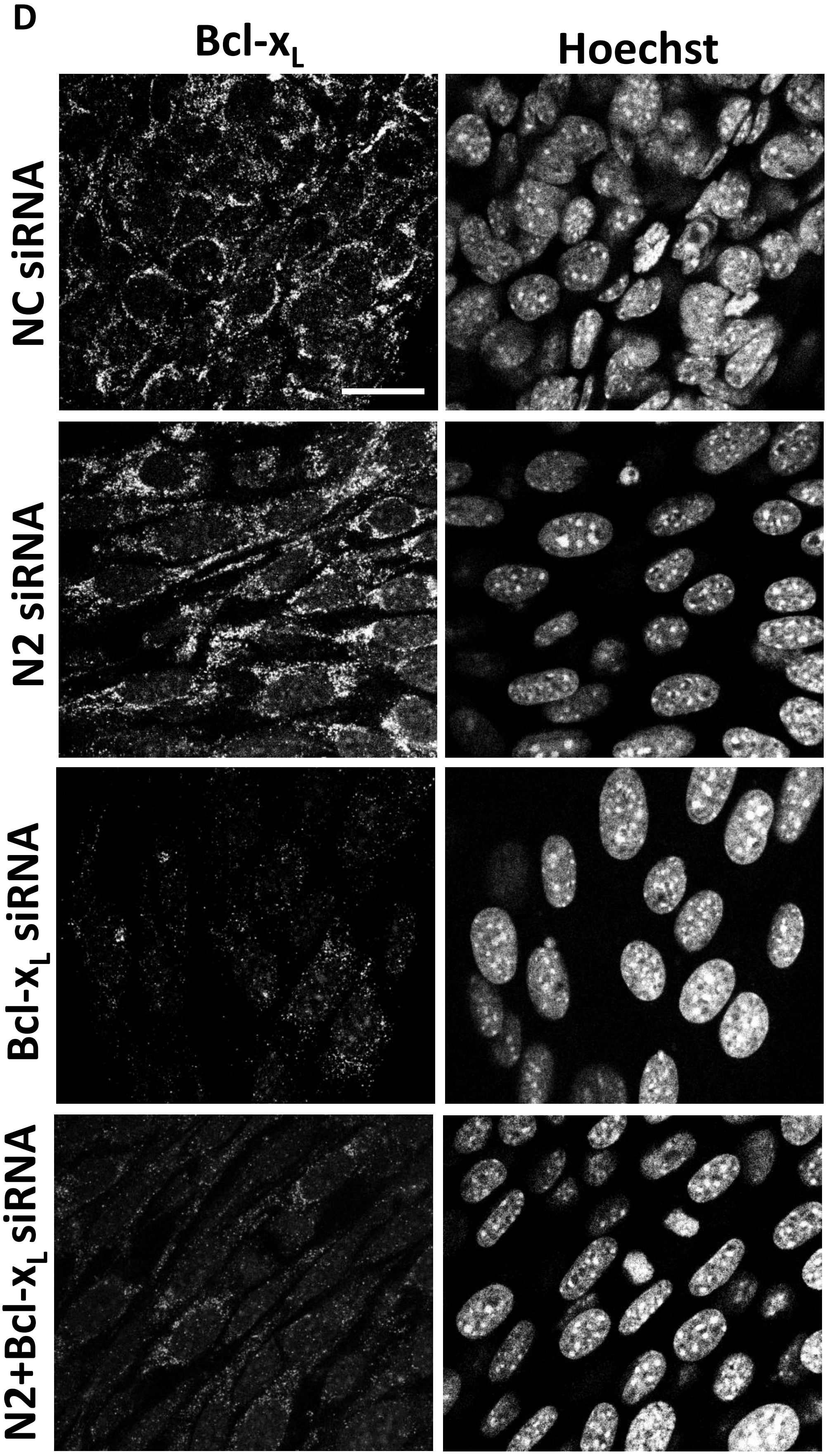

Figure S5

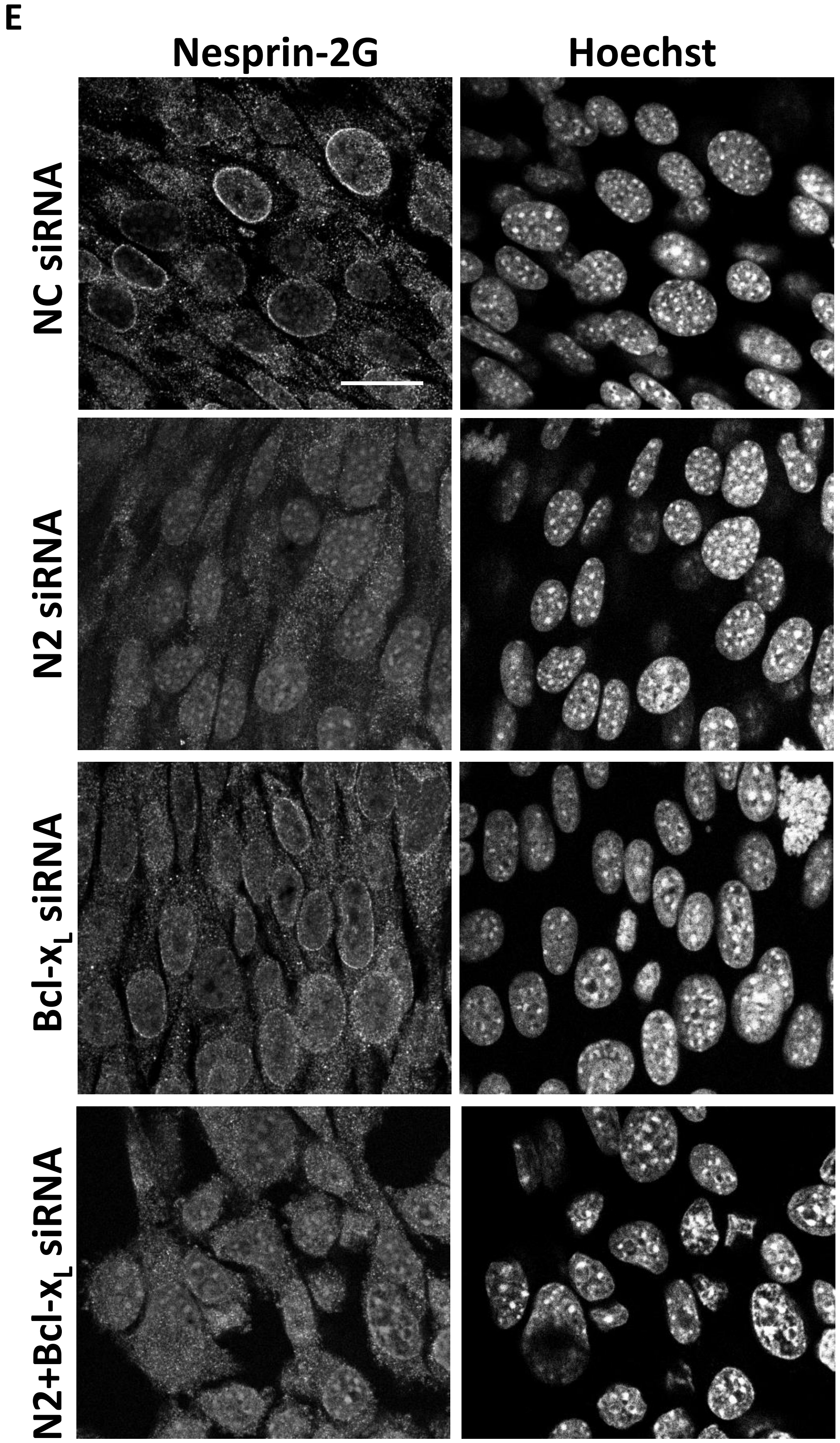
E
Nesprin-2G
Hoechst
NC siRNA
N2 siRNA
Bcl-x$_L$ siRNA
N2+Bcl-x$_L$ siRNA

Figure S5

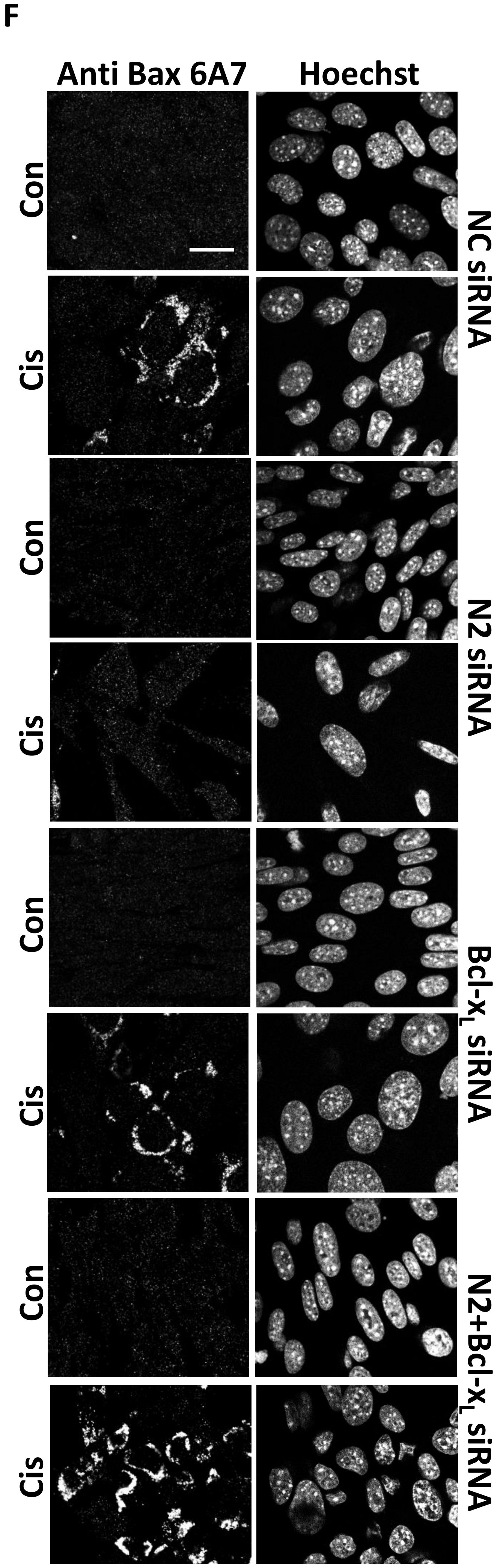
F
Anti Bax 6A7
Hoechst
Con
Cis
Con
Cis
Con
Cis
Con
Cis
NC siRNA
N2 siRNA
Bcl-x$_L$ siRNA
N2+Bcl-x$_L$ siRNA

Figure S5

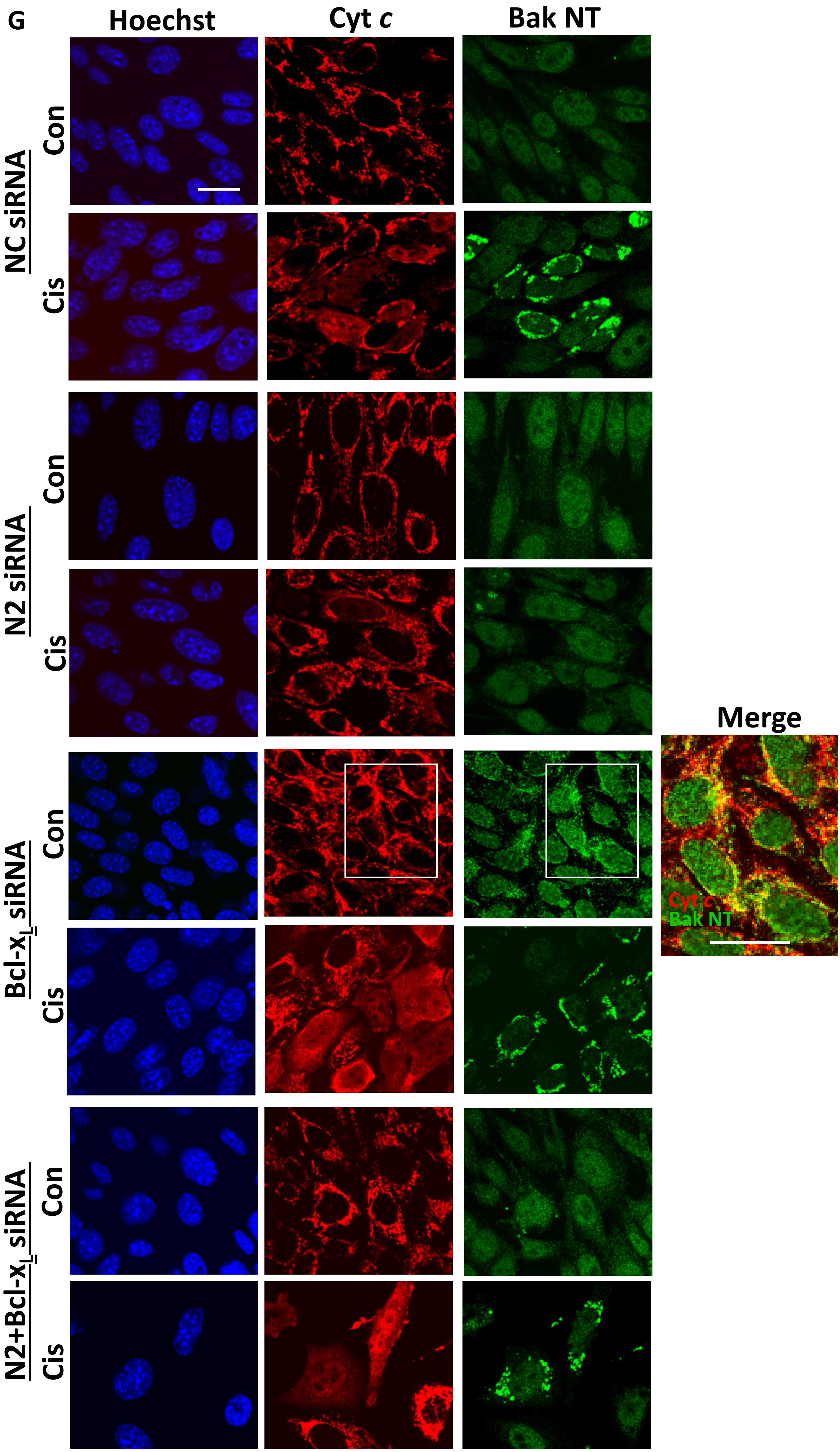
G
Hoechst
Cyt c
Bak NT
Merge
NC siRNA
Con
Cis
N2 siRNA
Con
Cis
Bcl-xL siRNA
Con
Cis
N2+Bcl-xL siRNA
Con
Cis
Cyt c
Bak NT

Fig. S5 Bcl-x_L is required for the nesprin-2 depletion survival effect. (A - C) Effects of nesprin-2 and nesprin-2G depletion in Bcl-x_L KO MEFs. (A, B) IF micrographs of Bcl-x_L KO MEFs untransfected (A) or transfected (B) with the indicated siRNAs and stained for anti-Bcl-x_L (A) or anti-nesprin-2G (B). In the latter (B) the left panels show the IF micrographs and the right panel shows quantification of the percentage of cells exhibiting NE-associated nesprin-2G in the indicated siRNA treatments (*n* = 3, 5 and 3 for NC siRNA, N2 siRNA and N2G siRNA, respectively). (C) Representative IF micrographs of Bcl-x_L KO MEFs transfected with the indicated siRNAs, treated with no drug (Con) or with cisplatin (Cis) and Q-VD-OPH and stained for Bax 6A7. The cells were treated and stained as described in Fig. 6C. Bar = 20 µm. (D - G) Effects of nesprin-2 depletion in WT MEFs depleted of Bcl-x_L. (D, E) Representative IF micrographs of WT MEFs transfected with non-targeting negative control (NC), nesprin-2 (N2), Bcl-x_L or nesprin-2 and Bcl-x_L (N2 + Bcl-x_L) siRNAs and stained for anti-Bcl-x_L (D) or anti-nesprin-2G (E). (F, G) Representative IF micrographs (from *n* = 3) of Bax 6A7 (F) or Bak NT and cytochrome *c* staining (G) in WT MEFs transfected with the indicated siRNAs and treated with no drug (Con) or with cisplatin (Cis) and Q-VD-OPH. The right panel in G is an enlarged merged image (cytochrome *c* and Bak NT staining) of the insert shown in the panel corresponding to con WT MEFs treated with Bcl-x_L siRNA. The cells were treated and stained as described in Fig. 6G. Bar = 20 µm.
