# Supplementary figures and images for: LINC complex protein nesprin-2 has pro-apoptotic activity via Bcl-2 family proteins
Source: Cell Death Discov. 2024 Jan 15;10:29. doi: 10.1038/s41420-023-01763-w (PMC10789774; doi:10.1038/s41420-023-01763-w)

Fig. 1

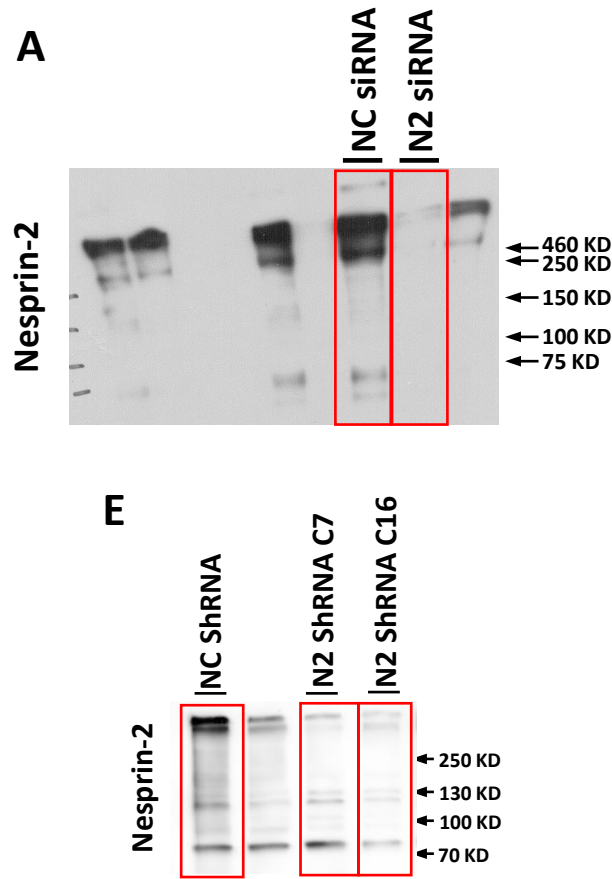

Fig. 2

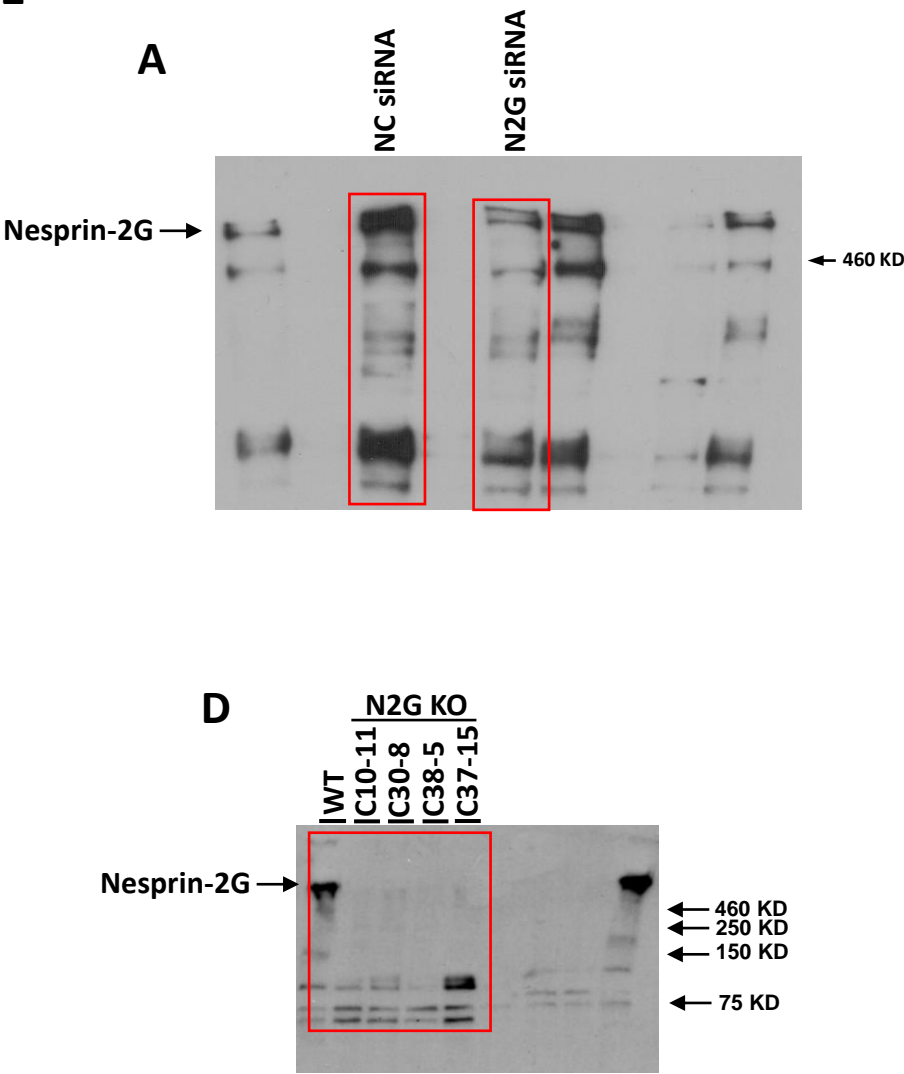

Fig. 3

A

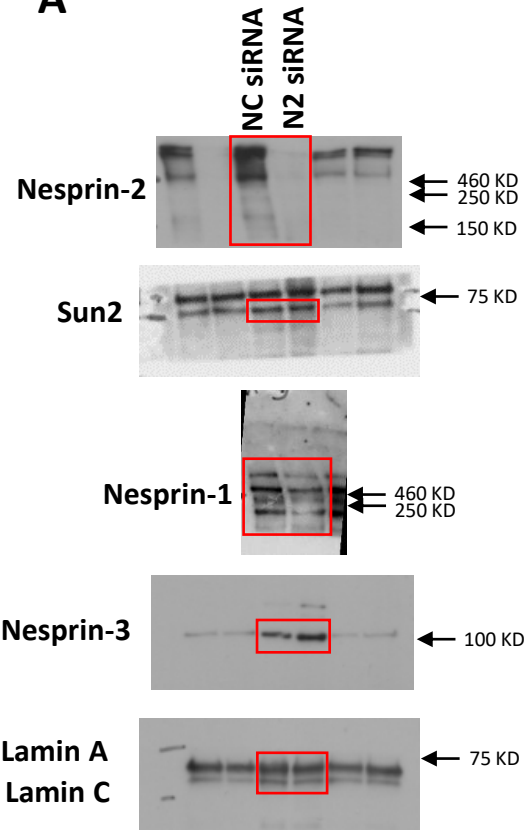

C

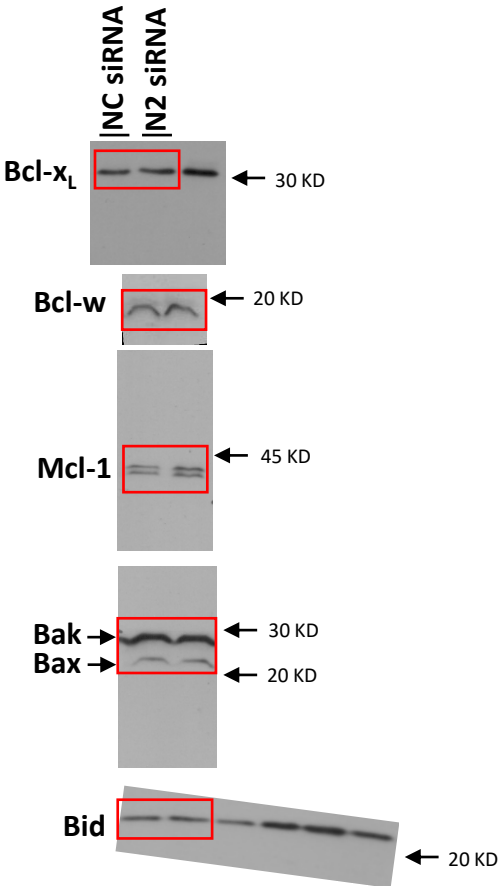

Fig. 6

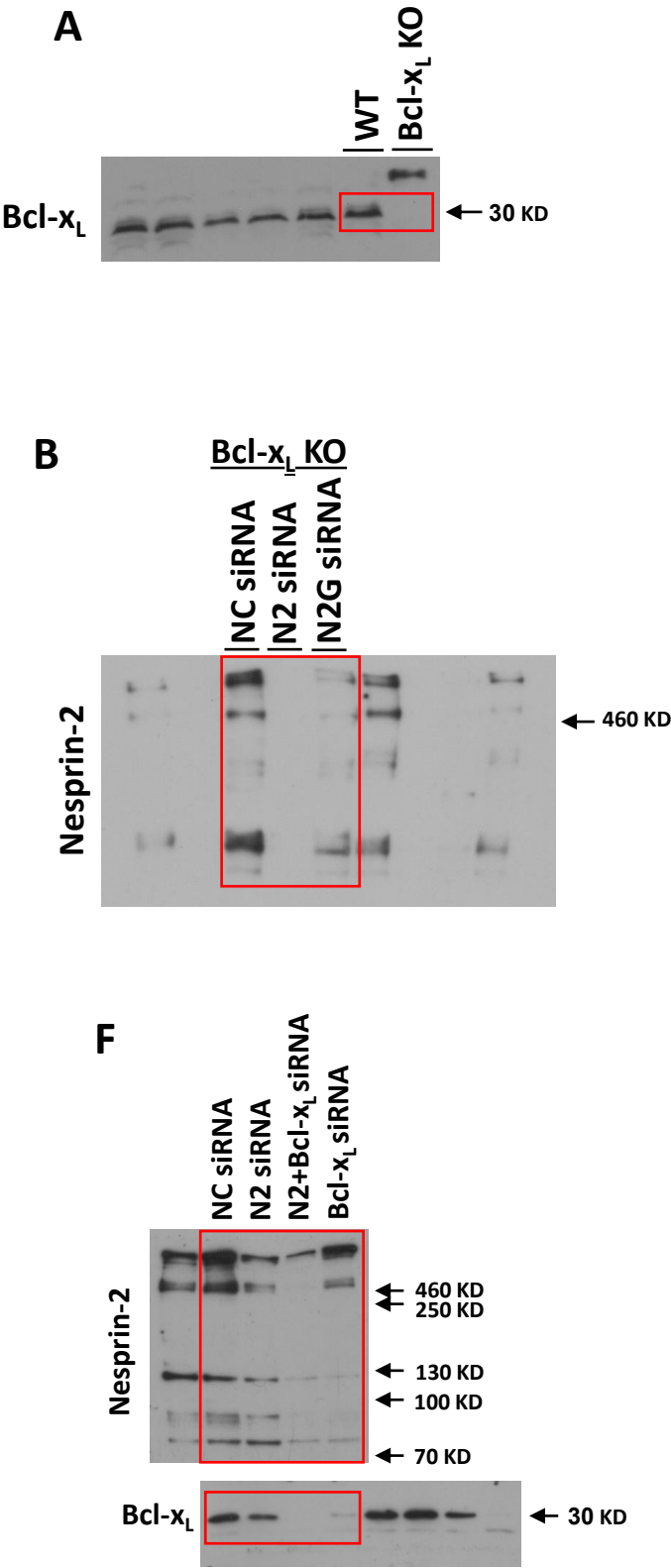

Fig. 7

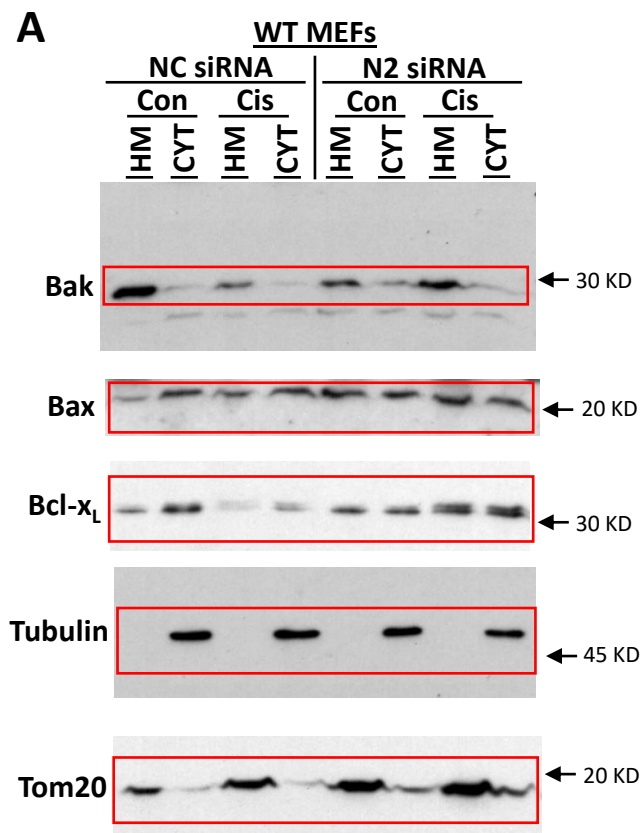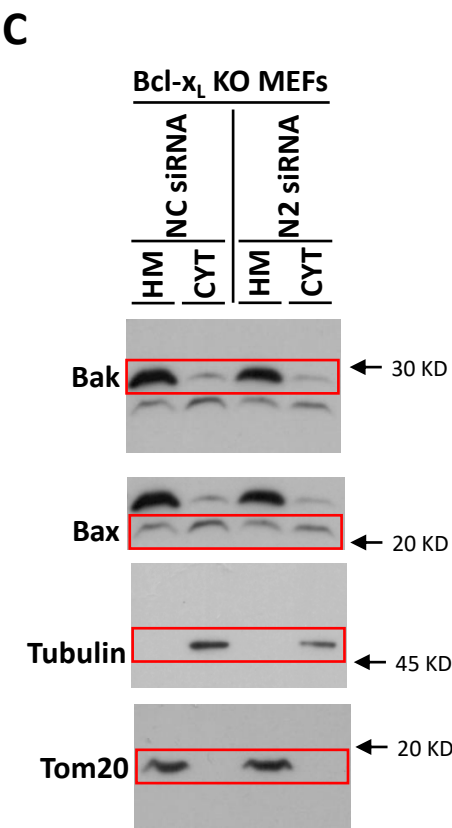

Supplement: Supplementary file 2 — Original Data File [file 41420_2023_1763_MOESM2_ESM.pdf]
